# A comprehensive study on engineering and sustainability characteristics with emphasizing on 3R's approach in building construction

**DOI:** 10.1016/j.heliyon.2024.e32206

**Published:** 2024-06-02

**Authors:** Samuvel Raj R, G. Prince Arulraj, N. Anand, Balamurali Kanagaraj, M.Z. Naser, Eva Lubloy

**Affiliations:** a Research Scholar, Department of Civil Engineering, Karunya Institute of Technology and Sciences, Coimbatore, India; b AI Research Institute for Science and Engineering (AIRISE), Clemson University, Clemson, SC 29634, USA; c Department of Construction Materials and Technologies, Budapest University of Technology and Economics, Budapest 1521, Hungary

**Keywords:** Sustainability, Geopolymer concrete, Signal-noise ratio, Grey relation analysis, Taguchi method, ANOVA

## Abstract

The study assesses the mechanical efficiency, long-lasting characteristics, microstructure, and sustainability of sustainable concrete (SC) samples through several optimization methods, emphasizing the significance of the 3Rs (recycle, reuse, reduce) approach in the construction sector. The study uses advanced techniques like the Taguchi method, grey relational analysis (GRA), analysis of variance (ANOVA), and signal-noise ratio (SNR) to optimize parameters affecting the performance of SC. In this study, the properties of SC are assessed by considering various parameters. These parameters include the use of 10 %, 20 %, and 30 % of ground granulated blast furnace slag (GGBFS) as a replacement for fly ash (FA). Additionally, six different binder contents ranging from 300 kg/m^3^ to 600 kg/m^3^ are examined. The study also investigates three different molarities of sodium hydroxide (NaOH) (8 M, 12 M, and 16 M), three different ratios of alkaline activators (AA) (1.5, 2.0, and 2.5), three different AA to-binder ratios (0.30, 0.35, and 0.40), and curing temperature (CT) of 30 °C, 60 °C, and 90 °C. The study includes fresh properties such as fresh density (FD) and slump, mechanical properties such as tensile strength (TS), flexural strength (FS), modulus of elasticity (MOE), and compressive strength (CS), and durability studies such as dry density (DD), impact strength, water absorption (WA), and sorptivity. The blended proportions were obtained using the Taguchi method. The study shows that GGBFS accelerates geopolymerization in FA-based concrete, reducing setting time and early-age CS. FA is crucial for setting time, workability, and CS enhancement. GGBFS increases the densities of fresh and hardened concrete, with a highly correlated increase, allowing accurate hardened density prediction with a coefficient of 0.9057. The CS of the cube SC surpassed 40 MPa, irrespective of variables such as the AA ratio, CT, and NaOH molarity. The trail mix with a binder concentration of 600 kg/m^3^, 30 % GGBFS content, 12 M NaOH molarity, 1.5 AA ratio, 0.35 AA to binder ratio, and 90 °C CT exhibited the greatest strength. Mixtures containing 10 % GGBFS can attain a CS above 30 MPa after 28 days, making them suitable for structural purposes. The T_18_ mix exhibited a compact Calcium (alumino) silicate hydrate (C-A-S-H) and N-A-S-H gel, whereas the T_3_ mix displayed a varied and permeable structure. The study used GRA, ANOVA, and SNR methods to analyze properties varying by six variables, finding GGBFS content as the most influencing parameter. The study found that the SC had a lower sustainability score than the OPC mix, but had better energy efficiency.

**Table:** 

List of Abbreviations			
AA	alkaline activators	IE	impact energy
ANOVA	analysis of variance	MLD	mixed level design
C-A-S-H	calcium alumino silicate hydrate	MOE	modulus of elasticity
C–S–H	calcium silicate hydrate	OPC	ordinary portland cement
CO_2_	carbon di oxide	RI	response index
CS	compressive strength	SEM	scanning electron microscopy
CT	curing temperature	SNR	signal-noise ratio
DD	dry density	N-A-S-H	sodium alumino-silicate hydrate
FA	fly ash	NaOH	sodium hydroxide
FD	fresh density	Na_2_SiO_3_	sodium silicate
FS	flexural strength	N–S–H	sodium silicate hydrate
GP	geopolymer	SC	sustainable concrete
GPC	geopolymer concrete	TS	tensile strength
GRA	grey relational analysis	WA	water absorption
GGBFS	ground granulated blast furnace slag		

## 1 Introduction

The increase in energy efficiency and the development of new building materials are both significantly influenced by sustainable development in both the building design and the civil infrastructure [[1], [2], [3], [4], [5], [6]]. Global warming and greenhouse gas emissions into the environment have been rising worldwide due to increased urbanization and population [7,8]. This adversely affects the globe, as it does in many industries, thus research on durable concrete production is attracting attention [9,10]. It is estimated that 1 tonne of carbon dioxide (CO_2_) is released into the environment to manufacture 1 tonne of ordinary portland cement (OPC) [11]. The sustainability of concrete can be improved by using substitute materials made from less energy energy-intensive techniques or recycled from other industrial operations [[12], [13], [14]]. The background of this research likely stems from the growing interest in sustainable construction materials and the need to reduce the environmental impact of traditional Portland cement-based concrete. FA and GGBFS are commonly used as supplementary cementitious materials due to their pozzolanic properties and their ability to reduce greenhouse gas emissions associated with cement production [[15], [16], [17], [18]]. GPC, which utilizes FA and/or GGBFS as binders, has emerged as a promising alternative to OPC concrete. However, optimizing the composition of GPC to achieve desired properties while minimizing costs and environmental impacts remains a challenge.

Compared to OPC, FA, GGBFS, and commercial by-products; release between 80 % and 90 % fewer greenhouse agents into the environment. In recent years, a substance known as geo-polymer (GP) categorized as a synthetic alumino-silicate, has been employed in place of cement-based binders in creating materials, including high-performance composites and ceramics. One of the most recent advancements in alternative cement research is unconsolidated GPs, which are created when alumino-silica compounds combine with alkali activators. Highly alkaline liquids must be combined with alumina-silicate reactive components to create GPs [19,20]. GPs are synthesized by aluminosilicate polycondensation. It is an aqueous alkali silicate liquid that has activated the minerals. GPs can be made using a variety of mineral and industrial by-products, including metakaolin, FA, and GGBFS, bagasse ash, bottom ash, rice husk ash, and pond ash [[21], [22], [23], [24], [25], [26], [27], [28], [29]]. GPs have a low-carbon footprint and hence serve as a sustainable alternative to traditional concrete [[30], [31], [32], [33]].

The hardened and fresh properties of GPC depend on many parameters. It is laborious and time-consuming to determine the effect of each parameter as the properties of GPC. The Taguchi approach, which reduces the number of combinations to be analyzed while taking into account each degree of the parameters to be examined, is inexpensive and quick. The Taguchi technique also offers a condensed explanation of how each variable degree affects the examined results in terms of characteristics [[34], [35], [36], [37], [38]].

The rationale behind this research is likely to explore the influence of various parameters on the properties of GPC. Understanding how parameters such as quantity of precursor, GGBFS content, NaOH molarity, AA ratio, and CT influence properties like slump, density, CS, FS, toughness, MOE, WA, and sorptivity which are crucial for developing high-performance GPC mixes. By identifying the optimal combination of parameters, researchers aim to improve the mechanical properties, durability, and sustainability of GPC, making it a viable alternative to conventional concrete in construction applications. A mathematical analysis technique based on the Taguchi and GRA is known as the Taguchi-based GRA method. The GRA technique helps scientists discover the numerical relationship between numerous elements which is a way to assess the correlation degree among different parameters following the similarity or difference degree of development trends among components. In this work, the Taguchi-GRA approach was used to examine the hardened and fresh characteristics of SC [39,40].

Nazari et al. [41] studied the design of CS of GP concrete made with cement as the alumina silicate source materials by the Taguchi method. They considered parameters such as water glass to NaOH ratio, concentration of NaOH, AA to binder ratio, CT, and time with four levels for each parameter. They found that the maximum 28-day strength of 43.1 MPa was obtained with the 14 M of NaOH, water glass to NaOH ratio of 1, 0.42 AA to binder ratio, and at ambient curing conditions. Riahi et al. [42] examined the mechanical strength of GP concrete cured by water using the Taguchi method. They considered three parameters CT and curing duration, and molarity of NaOH. Their findings show that concrete with a CT of 90 °C, molarity of NaOH of 8 M, and addition of rice husk ash and FA obtained maximum CS. Olivia and Nikraz [43] estimated the influence of aggregate content, activator-to-binder ratio, ratio of AAs, and curing type on the FA-based GP using the Taguchi method. Test results exhibited that; GP concrete had greater FS and TS, lesser shrinkage, and MOE than those of the OPC control mix. However, durability test results show that GP concrete is an alternative binding material for OPC concrete mainly in severe environments like sea water.

Mijarsh et al. [44] synthesized GP mortar using more amount of treated palm oil fuel ash. They studied the effect of additive materials and activator solutions on the GP mortar. Specimens with 65 % treated palm oil fuel ash and the remaining 35 % of other additives achieved maximum CS. The microstructural studies show that these specimens contain sodium alumina silicate hydrate (N-A-S-H) gel and calcium silicate hydrate (C–S–H) gel. Bagheri and Nazari [45] studied the mechanical strength of class C FA and GGBFS-based GP specimens using the Taguchi method by considering four different parameters such as GGBFS content, molarity of NaOH, curing time, and temperature. Results depicted that; the specimens with 12 M NaOH solutions, 30 % GGBFS content, and at 90 °C for 16 h provide good strength. They concluded that GGBFS added class C-based GP paste shows better early age strength. Mohammed et al. [46] designed a GGBFS-based GP composite reinforced with steel fiber. The water curing method, molarity of NaOH, ratio of activators to OPC content, and steel fiber content are the four different parameters that were considered for this study and the Taguchi method was used to design the experiments. Specimens with 14 M NaOH solution, activator to cement content ratio of 0.24, and 5 % steel content for 14 days of water curing show better TS.

Nazari and Sanjayan [47] examined the combined effects of crystalline nano alumina and amorphous nano silica on GP concrete specimens cured under water curing regimes at 2 and 7 days by using the Taguchi method. Results depicted that the WA depends upon the progression of the polymeric reaction. Specimens corresponding to the 90 °C of oven curing exhibited less WA percentage due to the denser GP paste due to the filling effect of nano materials. Panagiotopoulou et al. [48] used the Taguchi method to optimize the strength of GP concrete. They concluded that the optimum ratio of sodium to aluminium was 0.85 for maximum CS. Siyal et al. [49] studied the effects of four ratios of sodium and alumina, the ratio of alumina and silica, the ratio of water content to solid content, and CT on GP concrete by the Taguchi method. The Vicat apparatus results show the samples with 1.4 NA to Al_2_O_3_ ratio, 2.2 SiO_2_ to Al_2_O_3_ ratio, 0.30 water to solid ratio and temperature of 40 °C had higher setting time and workability. SiO_2_ to Al_2_O_3_ ratio of 1.8, NA to Al_2_O_3_ ratio of 1.0, water to solid ratio of 0.20, and 80 °C CT enhanced the setting of the GP specimens.

Ankur Mehta et al. [50] examined the effects of different parameters on FA-based GP concrete via the Taguchi statistical method. The addition of OPC, molarity of NaOH, and CT were the variable parameters considered for their study. The addition of OPC was found as the most influencing parameter for both CS and WA. Onoue and Bier [51] used a dynamic approach of the Taguchi method to optimize the GP concrete by considering different molarities of NaOH, the ratio of AAs, GGBFS content, mixing time, mixing method, cumulative temperature, and CT. Results show that a white patch formation was noticed after long-term immersion in a 10 % H_2_SO_4_ solution. Hadi et al. [52] designed GGBFS-based GP concrete at ambient curing conditions by using the Taguchi method. Binder content, AA to binder ratio, the ratio between sodium silicate (Na_2_SiO_3_) and NaOH, and molarity of NaOH solution were the variables considered for their study. They concluded that the partial replacement of GGBFS with FA, metakaolin, and silica fume shows better setting time. The highest CS was achieved with the specimen having 450 kg/m^3^ of binder content, 0.35 as the activators to binder ratio, 2.5 as the Na_2_SiO_3_ to NaOH ratio, and molarity of NaOH of 14 M. Dave and Bhogayata [53] optimized GP concrete mix design using the Taguchi method. These researchers developed specimens containing 14 M concentration of NaOH, 0.5 % superplasticizer content, the ratio of Na_2_SiO_3_ to NaOH of 2, and binder including 60 % FA, 30 % GGBFS, and 10 % silica fume exhibited greater strength.

Prusty and Pradhan [54] studied the influence of the water-to-solid ratio, NaOH concentration, binder content, and the ratio of AA on the GP concrete by using Taguchi GRA. Specimens with 55 % FA, 45 % GGBFS, a ratio between water to solid ratio of 0.31, molarity of NaOH of 14 M, a binder content of 450 kg/m^3,^ and a ratio of AA of 1.5 indicated better fresh as well as hardened properties. Chokkalingam et al. [55] investigated the mechanical and durability properties of GP concrete containing ceramic waste powder and GGBFS. Binder content, ceramic waste powder replacement by GGBFS, activators to binder ratio, the ratio of AAs, and the molarity of NaOH are the variables considered for their study. Specimens containing 450 kg/m^3^ of binder content, 60 % ceramic waste powder, 0.5 of activator to binder ratio, ratio of AA of 1.5, and 10 M concentration of NaOH achieved the maximum CS.

The scientific contribution of this study lies in its comprehensive investigation of the effects of multiple parameters on various properties of GPC. By utilizing the Taguchi method, GRA technique, and ANOVA, the study provides a systematic approach to optimizing the mix design and predicting the overall performance of GPC. Additionally, the study contributes to the understanding of the geopolymerization process, the role of supplementary materials like GGBFS, and the microstructural properties of hardened GPC through Scanning Electron Microscopy (SEM) analysis. Furthermore, comparing GPC with OPC concrete adds valuable insights into the environmental benefits of utilizing alternative binders for improving sustainability.

The study aims to determine the optimal control parameters for SC composition using the Taguchi-GRA approach. This involves assessing factors like binder content, GGBFS content, NaOH molarity, AA ratio, and CT to improve properties like slump, fresh and hardened density, CS, FS, TS, MOE, impact energy (IE), WA, and sorptivity. The study also investigates the effects of GGBFS on FA -based SC and its influence on setting time, early age CS, workability, and density. The study also examines the correlation between GGBFS content and properties like fresh and hardened concrete densities. The study uses analytical methods like GRA, ANOVA, and SNR to optimize parameter levels and develop regression equations. The results are validated with predicted outcomes to ensure accuracy. SEM analysis is conducted to examine the microstructure of optimized SC mixes and compare them with less optimal mixes. The sustainability index is evaluated to assess the environmental impact of SC compared to OPC mixes.

### 1.1 Research significance

**Fig. 1:**
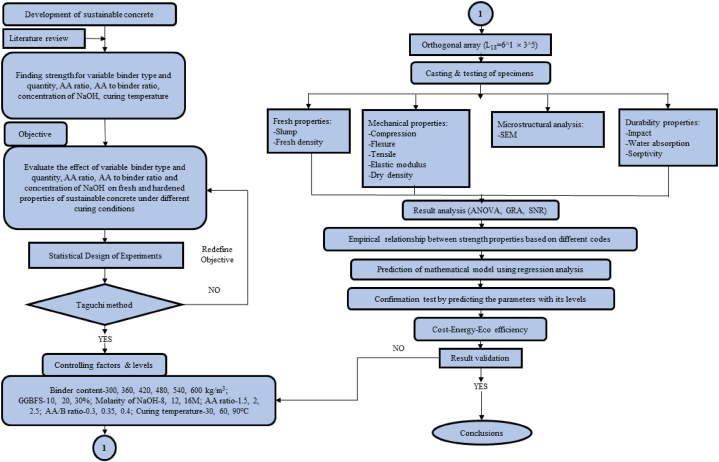
Schematic diagram of investigated research.

The literature lacks specific guidelines for developing SC considering parameters like binder proportions, AA concentration, curing conditions, alkaline-to-binder ratio, and AA ratio. To optimize variables, a non-expensive and time-efficient Taguchi GRA is used. The standard factorial method requires 1458 trials, while the Taguchi approach requires 18 trials. The mixed-level design (MLD) technique is used to construct the modified L_18_ orthogonal array, which is frequently used by the DOE for parameters with different quantities. The Taguchi approach uses the SNR for optimization. The study aims to optimize the influencing parameters of SC by studying properties at ambient curing conditions using the Taguchi method, ANOVA, and GRA. Regression equations and graphical explanations were obtained for all parameters. Sustainability was explored using different sustainability indexes. The ideal mix proportion was determined based on the SNR of specific characteristics using ANOVA and GRA. Fig. 1 shows a flow chart that summarises this study.

## 2 Materials and experimental methods

### 2.1 Materials

#### 2.1.1 Precursor materials

**Fig. 2:**
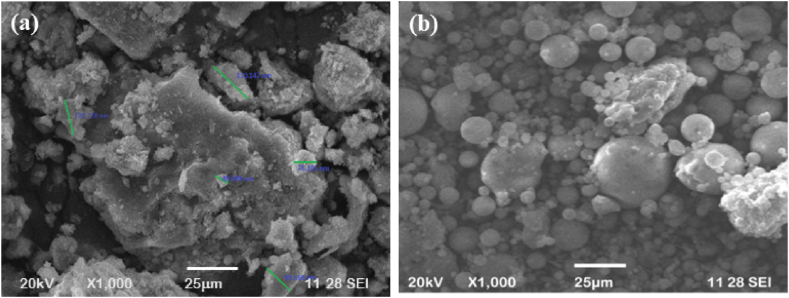
SEM image **(a)** GGBFS; **(b)** FA.

**Fig. 3:**
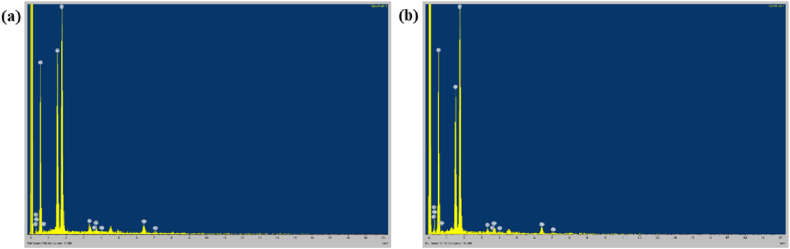
Edax of FA and GGBFS.

In this investigation, the binder used was class F FA or low calcium FA, which is collected from the Mettur Thermal power plant and confirms ASTM C 618 standards [56] and IS 3812:2013 [57]. FA was replaced with GGBFS as a binder material. Table 1 displays the characteristics and chemical compositions of FA (class F) and GGBFS as reported [58]. Additionally, the SEM micrograph and EDAX of FA and GGBFS are shown in Fig. 2 (a & b) and 3 (a & b), respectively. As observed, GGBFS particles are angular in shape, irregular, and amorphous. GGBFS is composed of quartz and gehlenite. The principal minerals found in the FA samples include quartz, anhydrite, lime, gehlenite, and calcite. SEM analysis revealed that the FA contains lignite combustion elements such as cenospheres (hollow spheres), planispheres, and agglomerates (see Fig. 3).

**Table 1:** Chemical composition of precursor materials [58].

Oxides	FA (%)	GGBFS (%)
SiO_2_	61.2	42.4
Al_2_O_3_	26.9	13.2
Fe_2_O_3_	6.21	1.12
CaO	1.91	41.2
MgO	0.29	1.3
Na_2_O	0.58	0.3
K_2_O	1.21	0.7
Ti	0.4	–

#### 2.1.2 Inert materials

**Fig. 4:**
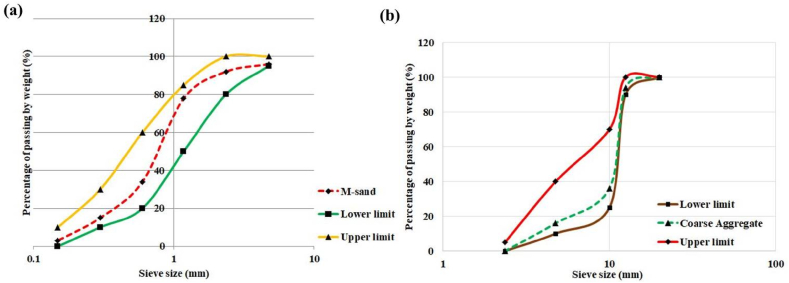
Particle size distribution curve for fine and coarse aggregate.

The fine aggregate utilized for this study has a maximum size of 4.75 mm and the coarse aggregate has a maximum size of 20 mm. Before mixing, all the aggregates were in a saturated surface dry condition. The fine aggregate utilized was M-sand. Both the aggregates conform to the guidelines of ASTM C33 [59]. The particle size distribution curves for M-sand and coarse aggregates are shown in Fig. 4 (a & b).

#### 2.1.3 Alkaline activators

NaOH and Na_2_SiO_3_ solutions of commercial grade (99 % purity) were utilized as activators in this study. The NaOH solution was created by mixing tap water with 97–98 % pure NaOH flakes to create NaOH solutions with molarities of 8 M, 12 M, and 16 M. The solution was then allowed to cool for at least 2 h before usage. To create the AA solution, NaOH and Na_2_SiO_3_ were mixed together.

### 2.2 Taguchi method and ANOVA

The Taguchi technique of analysis is used in the current study to identify the ideal design combination for SC [41,46,50,53,58,60]. Six key factors were taken into account when performing the Taguchi analysis: the binder content (300, 360, 420, 480, 540, and 600 kg/m^3^), the three different NaOH molarities (8, 12 and 16 M), the three different activators to binder ratios (1.5, 2.0, and 2.5), the three different AA to binder ratios (0.30, 0.35, and 0.40), and the CT of 60 °C and 90 °C and ambient curing condition. Table 2 tabulates the selected parameters and levels. Based on the L_18_ array obtained from the Taguchi technique [39], a total of eighteen trial mixes were prepared. Table 3 lists the design criteria for each of the trial mixes. Based on the SNR, the response index (RI) of each trial is determined [61]. The average 28th-day strength of each trial mix is taken into account to determine the RI of each particular variable.

**Table 2:** Variables and its magnitudes for the Taguchi technique of experimental design.

Parameters	Levels					
	A	B	C	D	E	F
Binder content (kg/m^3^)	300	360	420	480	540	600
GGBFS (%)	10	20	30			
Molarity of NaOH (M)	8	12	16			
AA ratio	1.5	2.0	2.5			
AA to Binder ratio	0.30	0.35	0.40			
Curing Temperature (^o^C)	30	60	90

**Table 3:** Experimental runs for the Taguchi method's L_18_ orthogonal array.

Trial Mix	Binder content (kg/m^3^)	GGBFS content (kg/m^3^)	Molarity of NaOH (M)	AA ratio	AA to Binder ratio	Curing temperature (^o^C)
1	300	10	8	1.5	0.3	30
2	300	20	12	2	0.35	60
3	300	30	16	2.5	0.4	90
4	360	10	8	2	0.35	90
5	360	20	12	2.5	0.4	30
6	360	30	16	1.5	0.3	60
7	420	10	12	1.5	0.4	60
8	420	20	16	2	0.3	90
9	420	30	8	2.5	0.35	30
10	480	10	16	2.5	0.35	60
11	480	20	8	1.5	0.4	90
12	480	30	12	2	0.3	30
13	540	10	12	2.5	0.3	90
14	540	20	16	1.5	0.35	30
15	540	30	8	2	0.4	60
16	600	10	16	2	0.4	30
17	600	20	8	2.5	0.3	60
18	600	30	12	1.5	0.35	90

The experimental design for assessing the properties of SC based on FA-GGBFS involved varying factors such as GGBFS replacement level, AA ratio, binder content, molarity of NaOH solution, AA to binder ratio, and CT. The study selected six levels for binder content and three levels for the other parameters. Using the full factorial design technique, a total of 1458 experimental permutations (i.e., Level^Parameter^: 6^1 × 3^5) would be required to analyze the influence of each parameter, making it a time-consuming and inefficient process [54]. To address this, the Taguchi method was employed to examine the effects of mixed variable levels on SC properties with fewer experiments, determining the optimal combination of factors for the best results. The Taguchi method's L_18_ orthogonal array was used, and the selected experimental runs are presented in Table 3.

The gathered results are converted into the SNR using the Taguchi method of experiment design. There are 3 functions: “larger the better”, “nominal the better” and “smaller the better”, and one of them must be chosen depending on the conditions and the various characteristics (responses) [39,40]. The SNR of the reaction was calculated based on the “larger-the-better” function since higher workability and higher mechanical properties are required of FA-GGBFS-based SC while lower WA and rate of WA are required, the SNR is calculated based on the “smaller-the-better” principle using the following Eqs. (1) and (2).

(1) $$X_{ij}=-10\;\log_{10}\left[\frac{1}{n}\sum_{k=1}^{n}\frac{1}{y_{ijk}^{2}}\right]$$

(2) $$Y_{ij}=-10\;\log_{10}\left[\frac{1}{n}\sum_{k=1}^{n}y_{ijk}^{2}\right]$$

Here, X_ij_ and Y_ij_ stand for the SNR of the ith experiment for the jth response, and y_ijk_ represents the outcome of the ith experiment for the jth response in the kth replication and n is the number of replications.

The study used ANOVA to analyze the impact of response parameters on mechanical and fresh properties. The F-value was calculated by dividing the sum of squares of the studied parameter by the sum of squares of all other parameters. The mean SNR for each response or property level was also determined to determine the ideal combination of parameters for specific characteristics.

### 2.3 Grey relation analysis

A unique best-setting combination of the variables concerning numerous responses was obtained using the Taguchi-GRA method [39,47]. First, using Eqs. (3) and (4), each response's SNR was converted into a normalized value.

(3) $$Z_{ij}=\frac{X_{ij}-\min\left(X_{ij}\right)}{\max\left(X_{ij}\right)-\min\left(X_{ij}\right)}$$

(4) $$Z_{ij}=\frac{Y_{ij}-\min\left(Y_{ij}\right)}{\max\left(Y_{ij}\right)-\min\left(Y_{ij}\right)}$$

Z_ij_ = normalized SNR of ith experiment for jth response. Subsequently, the following equation (Eq. (5)) was used to get the grey relationship coefficient.

(5) $${GRC\;}_{0ij}=\frac{\Delta_{\min}+\zeta \;\Delta_{\max}}{\Delta_{0ij}+\zeta \;\Delta_{\max}}$$

Δ_0ij_ = difference between the ideal value of the normalized SNR (Z_0j_) and the normalized SNR (Z_ij_). Eqs. (6) and (7) is used to calculate $\Delta_{\min}$ and $\Delta_{\max}$.

(6) $$\Delta_{\min}=\min_{\forall i}\;\min_{\forall j}\Delta_{0ij}$$

(7) $$\Delta_{\max}=\max_{\forall i}\;\max_{\forall j}\Delta_{0ij}$$

ζ = Identification or differentiating coefficient (values range from 0 to 1 and it is usually set as 0.5). The equation below (Eq. (8)) was employed to determine the grey relational grade.

(8) $${GRG}_{0j}=\sum_{j=1}^{n}\omega_{j}{GRG}_{0ij}$$

GRG_0i_ = grey relational grade of ith experiment; ω_j_ = normalized non-negative weight assigned to jth response, and $\sum_{j=1}^{n}\omega_{j}$ = 1 [39,40]. Equal weight was given to each property in the current study.

### 2.4 Mix proportion of the specimens

Table 4 shows mix proportions of SC mixes, with 20 mm and 10 mm aggregates combined in 60 % and 40 % ratios. Alkaline liquid content was determined by using the Taguchi method based on NaOH solution molarity, AA to Binder ratio, and binder content.

**Table 4:** Mix proportion of the trail mixes.

Trail Mix	Binder (kg/m^3^)	GGBFS replacement level (%)	AA/B ratio	Binder (kg/m^3^)		FA (kg/m^3^)	CA (kg/m^3^)		AA ratio	AA (kg/m^3^)		Molarity of NaOH (M)	Curing temperature (^o^C)
				FA	GGBFS		10 mm	20 mm		NaOH	Na_2_SiO_3_		
1	300	10	0.3	270	30	770.50	594.39	891.58	1.5	28.80	43.20	8	30
2	300	20	0.35	240	60	724.30	558.75	838.12	2	50.40	100.80	12	60
3	300	30	0.4	210	90	655.70	505.83	758.74	2.5	76.80	192.00	16	90
4	360	10	0.35	324	36	722.44	557.31	835.97	2	40.32	80.64	8	90
5	360	20	0.4	288	72	651.88	502.88	754.32	2.5	69.12	172.80	12	30
6	360	30	0.3	252	108	692.20	533.98	800.97	1.5	69.12	103.68	16	60
7	420	10	0.4	378	42	655.90	505.98	758.97	1.5	80.64	120.96	12	60
8	420	20	0.3	336	84	632.38	487.84	731.75	2	80.64	161.28	16	90
9	420	30	0.35	294	126	677.46	522.61	783.92	2.5	47.04	117.60	8	30
10	480	10	0.35	432	48	534.48	412.31	618.47	2.5	107.52	268.80	16	60
11	480	20	0.4	384	96	664.40	512.54	768.81	1.5	61.44	92.16	8	90
12	480	30	0.3	336	144	633.04	488.35	732.52	2	69.12	138.24	12	30
13	540	10	0.3	486	54	575.74	444.14	666.21	2.5	77.76	194.40	12	90
14	540	20	0.35	432	108	558.10	430.53	645.80	1.5	120.96	181.44	16	30
15	540	30	0.4	378	162	613.54	473.30	709.95	2	69.12	138.24	8	60
16	600	10	0.4	540	60	446.20	344.21	516.32	2	153.60	307.20	16	30
17	600	20	0.3	480	120	597.40	460.85	691.28	2.5	57.60	144.00	8	60
18	600	30	0.35	420	180	568.00	438.17	657.26	1.5	100.80	151.20	12	90

### 2.5 Mixing, casting, curing, and analysis of the results

**Fig. 5:**
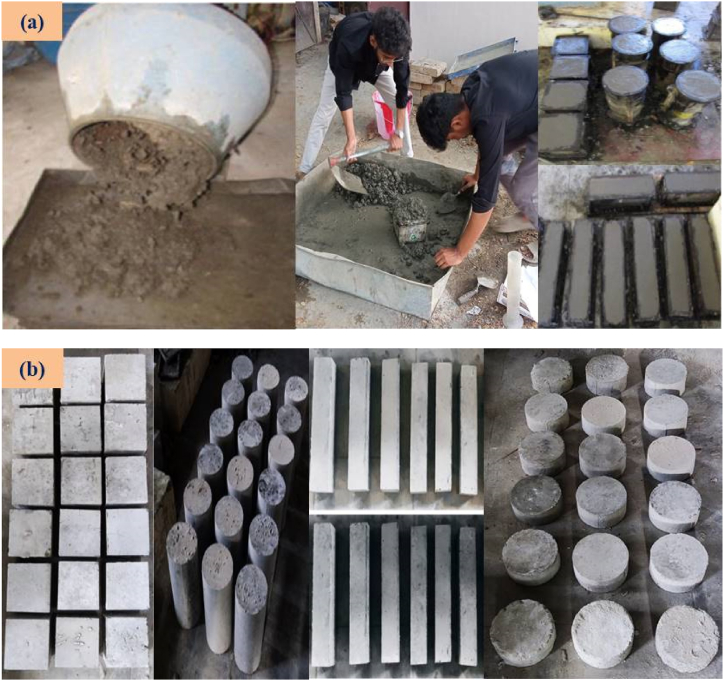
(a) Mixing and Casting of SC specimens; (b) Curing of SC specimens.

**Fig. 6:**
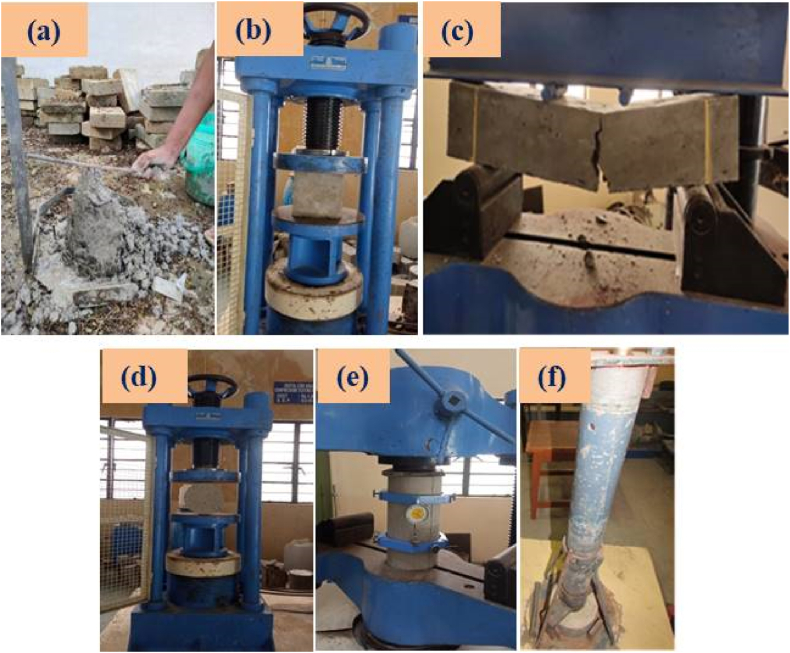
(a) Slump test; (b) Compression test; (c) Flexural test; (d) Splitting tensile test; (e) MOE test; (f) Impact test.

The preparation of SC involves dry-mixing fillers, binder materials like FA and GGBFS, and producing an AA solution. The dry ingredients are whirled in a mixer drum for 2–3 min, then the AA solution is added and the mixer drum rotates for an additional 2 min until the desired consistency is reached. The process continues until the desired consistency is achieved. In this work, the mechanical properties of SC are obtained using three distinct sets of moulds. The concrete specimens were cast in 150 mm × 150 mm x 150 mm cube moulds, 500 mm × 100 mm x 100 mm prism moulds, and 150 mm diameter and 300 mm length cylindrical moulds to evaluate the mechanical characteristics of SC. To remove air bubbles, moulds were filled with SC and vibrated for 10–15 s. The specimens were allowed to rest for 24 h at room temperature. After being removed, they were cured at 60 and 90 °C for 24 h. The specimens to be cured under ambient conditions were kept in the room until testing. The SC mixing, castings, and, curing, are shown in Fig. 5 (a & b). The different properties of sustainable concrete, tests conducted for this study, and the standard code details are presented in Table 5. The SC testing images are shown in Fig. 6 (a – f).

**Table 5:** Experimental details.

Properties	Tests	Standard code
Fresh concrete	Initial setting time	ASTM C 191 (2008) [62]
	Final setting time	ASTM C 191 (2008) [62]
	Slump	ASTM C 143 [63]
Hardened concrete	Compressive strength	IS 516 (2004) [64]
	Tensile strength	ASTM C 496 (2011) [65]
	Flexural strength	ASTM C 78 (2002) [66]
	Modulus of elasticity	ASTM C469 [67]
	Dry density	ASTM C 138, 2017
Durability properties	Impact strength	ACI 544 [68]
	Water absorption	ASTM C 1585 (2004) [69]
	Sorptivity	ASTM C1585-13 (2004) [70]
Microstructural properties	SEM	–
Sustainability properties	Cost efficiency	–
	Energy efficiency	–
	Carbon efficiency	–

## 3 Results and discussion

### 3.1 Taguchi method

#### 3.1.1 Slump

**Fig. 7:**
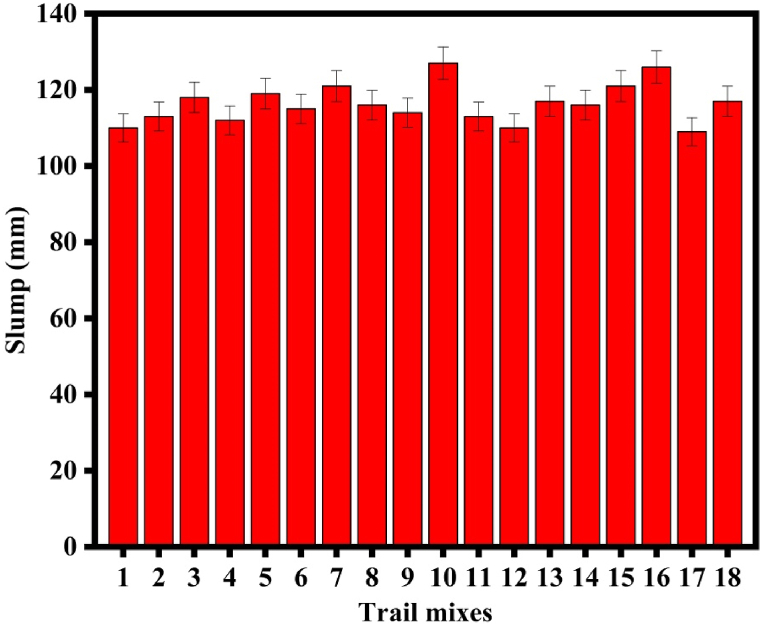
Slump plot for the specimens.

Fig. 7 depicts the workability towards FA replacement with various GGBFS proportions and other influencing factors. From Table 4 and Fig. 7, it is clear that the AA-to-binder ratio and GGBFS content had an impact on the slump of the SC mix. Trials with the highest AA to-binder ratio and 30 % GGBFS exhibit the poorest slump, an increase in GGBFS content greatly reduces the workability. The lowest slump value is provided by the T_13_ mix. The less mobility FA and GGBFS particles are most likely responsible for the mix's decreased slump. Due to the high reactivity of GGBFS, which consumes the water content resulting reduces the setting time of the concrete as well as reduces the workability of the whole system. Therefore, the AA to binder ratio is directly proportional to the slump value whereas; GGBFS content is inversely proportional to the slump of SC [52]. The findings lead to the hopeful conclusion that FA-GGBFS-based SC can achieve reasonable workability.

FA is a pozzolanic material that reacts with calcium hydroxide and water in the presence of cementitious materials like Portland cement to form additional cementitious compounds. This pozzolanic reaction is slower than the hydration of cement, extending the overall setting time of the concrete. The delayed pozzolanic reaction may delay the initial strength gain of the concrete mixture, as it takes longer to achieve significant CS compared to a mixture without FA. However, the long-term strength development of the concrete can be enhanced by the presence of FA due to the continued pozzolanic reaction over time. FA also reacts with alkalis in the presence of water to form additional cementitious compounds, contributing to the setting and hardening of the alkali binder paste. The alkali content of the activator solution used to mix with FA can influence the setting time, with higher concentrations generally promoting a faster setting due to increased reactivity with FA particles. The proportion of FA in the alkali binder paste mixture can also impact the setting time, with warmer temperatures generally accelerating the setting process and cooler temperatures slowing it down.

#### 3.1.2 Fresh and dry density

At both the hardened and fresh states, the density of SC mixtures proportioned with varied binder contents and other different influencing parameters were investigated. The fresh condition generally had the maximum density, with values between 2267 kg/m^3^ and 2491 kg/m^3^. The density dropped between 0.22 and 3.24 % after 28 days of curing. The greatest hardened density of 2460 kg/m^3^ was achieved by Mix 15, which had 540 kg/m^3^ of binder, 30 % GGBFS, AA to binder ratio of 0.40, Na_2_SiO_3_ to NaOH ratio of 2.0, and NaOH solution molarity of 8 M. It was cured at a temperature of 60 °C [71]. Notably, mixes with binder contents between 480 kg/m^3^ and 600 kg/m^3^, GGBFS replacement of 30 %, AA to Binder ratio of 0.30–0.40, Na_2_SiO_3_ to NaOH of 1.5–2.0, NaOH solution molarity of 8 M–12 M, and CT between 60 °C and 90 °C as well as room temperature could achieve hardened densities greater than 2400 kg/m^3^. On the other hand, Biricik's [33] results illustrate the change in unit weight of GPMs over time and under different curing conditions. They observed a decrease in unit weight under air and laboratory curing conditions for GPMs with certain Blaine fineness values, while water curing led to an increase in unit weight. The regression equations derived from their data allowed the estimation of CS based on the change in unit weight, with high degrees of regression. Comparing the two studies, both explored the effects of different parameters and curing conditions on the density or unit weight of geopolymer materials. While our study focuses on the density of SC mixtures, Biricik's study examines the change in the unit weight of GPMs. Both studies provide valuable insights into optimizing geopolymer formulations and curing methods to achieve desired density or unit weight properties.

**Fig. 8:**
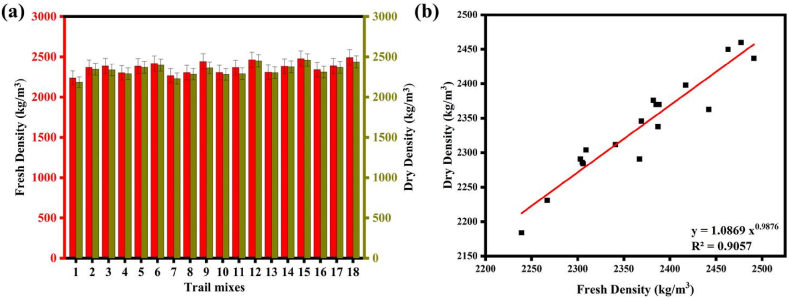
(a) Fresh and DD of SC; **(b)** Correlation between the fresh and DD of SC.

Fig. 8(a) shows the fresh and DD of SC specimens and Fig. 8(b) shows a scattered diagram between FD and DD. The results show that it is possible to forecast the SC's 28-day DD from its FD. Eq. (12) in Fig. 8(b) illustrates the correlation between FD and DD (R^2^ = 0.9057).

(12) $$DD=1.0869{FD}^{0.9876}$$

#### 3.1.3 Compressive strength

**Fig. 9:**
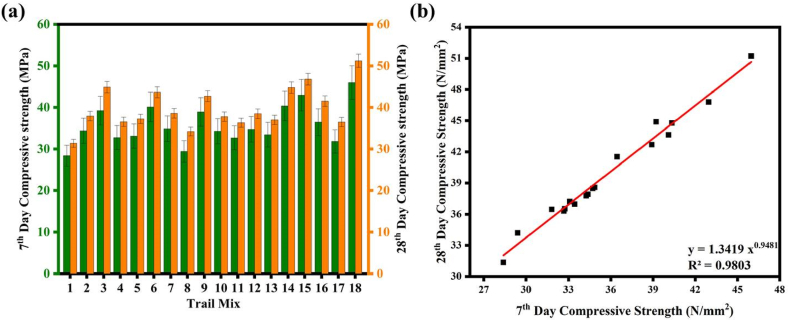
(a) CS of SC; **(b)** Correlation between 7th day and 28th day CS.

At the 7th, and 28th days of curing, the CS of GP concrete mixtures made with various GGBFS replacement levels is displayed in Fig. 9(a). Given that there was not much difference in the CS trends between the 7th and 28th days, the discussion will concentrate on the 28th day CS. The SC mixtures were categorized according to their FA replacement level with GGBFS. For 20 % and 30 % GGBFS replacement categories, mixes 2, 3, 5, 6, 8, 9, 11, 12, 14, 15, 17, and 18 showed the highest 28th-day CS than the specimens with 10 % GGBFS replacement category mixes 1, 4, 7, 10, 13 and 16. Among the 30 % replacement category, the specimens T_15_ and T_18_ had the max strength of 46.80 MPa and 51.22 MPa, respectively. The concentration of CaO enhanced when the amount of GGBFS increased in the mix, which may account for the 38 % increase in CS of SC.

The increased levels of free CaO in the mixture speed up the polymerization reaction, resulting in the formation of C-A-S-H gel. Their binder content, GGBFS replacement percentage, AA to Binder ratio, Na_2_SiO_3_ to NaOH ratio, NaOH solution molarity concentration, and CT appear to be in the range of 540 kg/m^3^ to 600 kg/m^3^, 30 %, 0.35–0.40, 1.5–2.0, 8 M–12 M and 60 °C–90 °C respectively. According to Fig. 9 observations, the CS increases with increasing molar concentration of NaOH solution. Higher OH ions may be responsible for the interaction with the FA and GGBFS material to produce more polymeric gel. Furthermore, increasing the molarity of the NaOH solution resulted in stronger and more rapid hydroxide ion dissolution is evident from the investigation that NaOH with a larger molar concentration retains more CS at a particular level beyond that limit there will be a marginal increase in the CS. On the other side, the low AA to Binder enhanced the CS, decreased the void content, and improved particle packing density [72,73].

Furthermore, a higher 28th-day CS was produced by adding more GGBFS to SC mixes. This enhancement could be attributed to the material's granular structure and stronger hydraulic response capabilities, which facilitated an accelerated reaction of calcium and the development of gels known as C–S–H and C-A-S-H [74]. As seen in the density and SEM data, a denser matrix is responsible for this performance improvement. Lower Na_2_SiO_3_ to NaOH ratios and ambient curing conditions of the FA-GGBFS blended GP concrete could result in equivalent CS greater than 35 MPa [75]. The strength grew by 9.5 % between 7 and 28 days, showing that the majority of the reaction occurred in the first 7 days. This is explained by the GGBFS's rapid reaction with high NaOH molarity, which results in the formation of C-A-S-H and C–S–H gels [76]. The corresponding strength gains from 7 to 28 days ranged from 3.3 % to 74.1 %. Due to FA's slower activation reaction under ambient circumstances, mixtures with 30 % GGBFS substitution had the greatest improvement in strength. All trial mixtures had a minimum CS of 40 MPa. Similarly, Biricik's [33] results show that GPMs with higher Blaine fineness values exhibited higher CS. Specifically, M − 6000W GPMs cured in water-saturated lime at 22 ± 2 °C exhibited the greatest CS of 63.6 MPa at 56 days. The study also observed a significant increase in CS with decreasing particle size of GGBFS. Furthermore, both studies observed the influence of activator parameters such as NaOH solution molarity concentration on CS. Higher concentrations of NaOH solution led to stronger and more rapid dissolution of hydroxide ions, resulting in higher CS up to a certain limit.

The results show that it is possible to forecast the SC's 28th day CS from its 7th day CS. Eq. (13) in Fig. 9(b) illustrates the correlation between 7th day and 28th day CS (R^2^ = 0.9803).

(13) $${CS}_{28-day}=1.3419{CS}_{7-day}^{0.9481}$$

#### 3.1.4 Tensile and flexural strength

The T_18_ trial mixes with 30 % FA substitution by GGBFS, an AA ratio of 1.5, and a NaOH concentration of 12 M had the highest TS and FS. The polymerization reaction process was accelerated by the high calcium oxide concentration in GGBFS. This may be explained by the calcium oxides' release of heat, which contributes to creating a strong polymeric chain and, as a result, helps to generate C–S–H gel together with Sodium Silicate Hydrate (N–S–H) and N-A-S-H gel [53]. On the other hand, the T_1_ specimens with an 8 M concentration of NaOH, an AA ratio of 1.5, and a binder made solely of FA 90 % and GGBFS 10 % had the poorest TSs and FSs. Similarly, Biricik's [33] results show that GPMs with higher Blaine fineness values exhibited higher FS. For instance, M − 6000W GPMs cured in water-saturated lime at 22 ± 2 °C exhibited the greatest FS of 7 MPa at 56 days after production. The study also observed significant increases in FS with increasing curing time. Furthermore, both studies discuss the mechanism of geopolymerization, which involves ion exchange, hydrolysis, network breakdown, release of Si and Al, convergence, gelation, re-organization, and geopolymerization. Understanding this mechanism is crucial for predicting FS based on changes in unit weight.

**Fig. 10:**
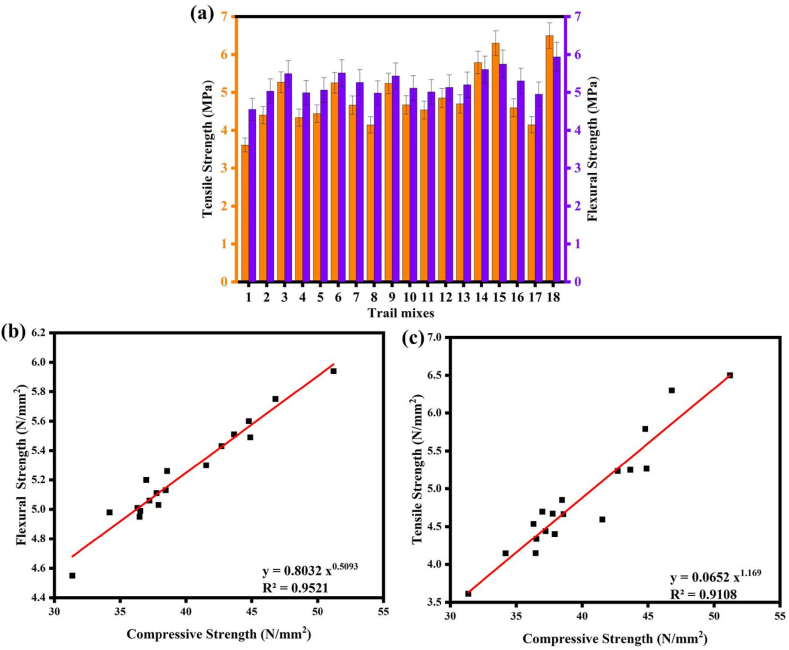
(a) FS and TS of SC; **(b)** correlation between FS and CS; **(c)** correlation between TS and CS.

Fig. 10(a), shows the results of the TS and FS. The FS can be predicted from CS with a high correlation coefficient (R^2^ = 0.9521) is illustrated in Fig. 10(b). A relationship between the two properties was developed using linear regression analysis and is given in Eq. (14) and Eq. (15). It is feasible to forecast either TS from CS or vice versa with a strong correlation coefficient (R^2^ = 0.9108) is shown in Fig. 10(c).

(14) $$FS=0.8032{CS}^{0.5093}$$

(15) $$TS=0.0652{CS}^{1.169}$$

#### 3.1.5 Modulus of elasticity

**Fig. 11:**
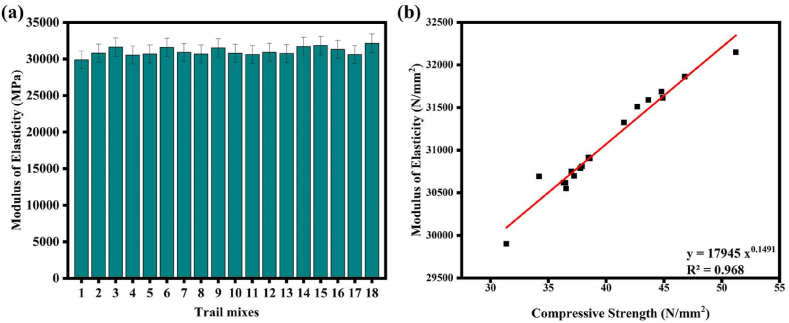
(a) MOE of SC; **(b)** correlation between MOE and CS.

Fig. 11(a) shows the curves for the MOE of GP concrete mixtures. The maximum MOE was exhibited by mixes 15 and 18, which contained 30 % GGBFS replacement and had elasticity values of 31864 N/mm^2^ and 31741 N/mm^2^, respectively. Their corresponding binder content, AA to Binder, Na_2_SiO_3_ to NaOH ratio, NaOH solution molarity, GGBFS content, and CT were given the values separated for the mixes 15 and 18 540 kg/m^3^ to 600 kg/m^3^, 0.35 to 0.40, 1.5 to 2.0, 8 M–12 M, and 30 %, respectively. Fig. 11(a) illustrates the impact of the replacement of FA with GGBFS on the elasticity modulus of SC. The mix with the highest MOE at 30 % GGBFS replacement level was mix 18.

From the scatter plot of Fig. 11(b) a linear regression equation was developed and is given in Eq. (16). It can predict the MOE from CS with a high correlation coefficient (R^2^ = 0.968).

(16) $$MOE=17945{CS}^{0.1491}$$

#### 3.1.6 Impact energy

**Fig. 12:**
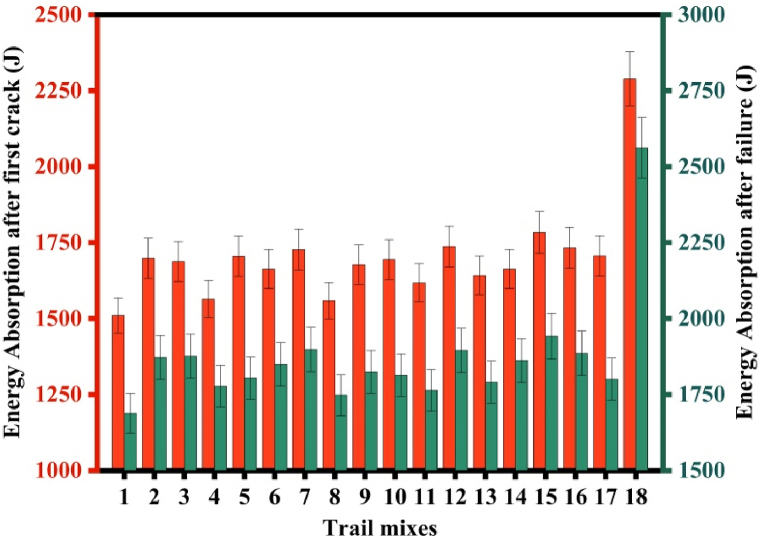
Energy absorption for the specimens.

**Fig. 13:**
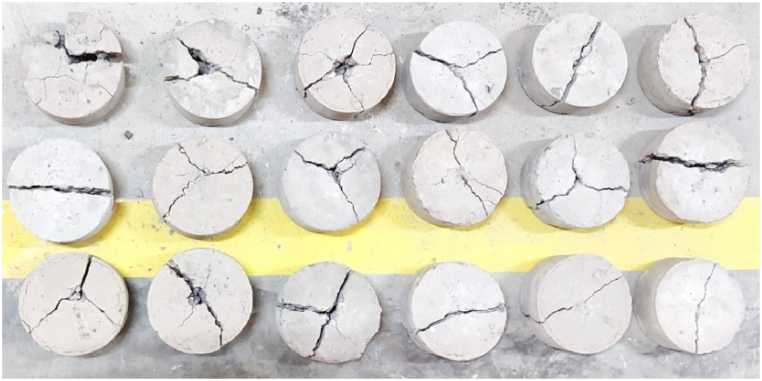
Failure pattern of the specimens.

On SC discs exposed to impact stress, the total number of blows required for the initial and final cracks were observed. The specimens' energy absorption is calculated by using the ACI 544.2 R-89 equation [68]. As shown in Fig. 12, the impact resistance was found to improve as GGBFS content increased up to a replacement level of 42 %. For the initial crack, the increase in resistance of the mix with 10, 20, and 30 % GGBFS was found to be 2, 13, and 34 % respectively. But, in the case of the final crack, the increase in resistance was found to be 11, 20, and 42 %. Mix blended with GGBFS has the capacity to withstand the impact load. This can be explained by the increase in energy absorption resistance, binder materials like GGBFS and FA have high-stress distribution mechanisms and adequate molecular attachment capabilities between traditional filler materials. The failed samples displayed that, the final cracking across the GP binder gel, which is shown in Fig. 13.

#### 3.1.7 Water absorption and sorptivity

**Fig. 14:**
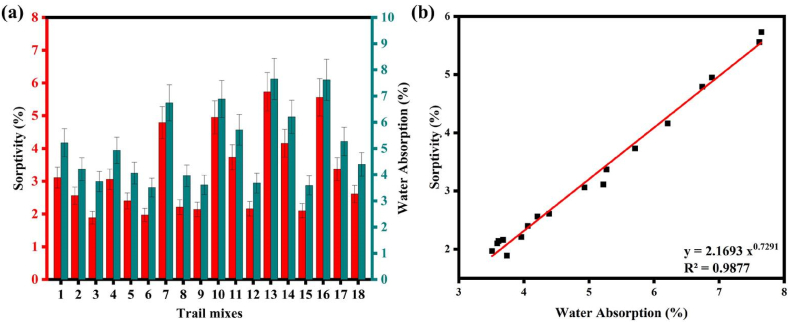
(a) WA and sorptivity of SC; **(b)** Correlation between WA and sorptivity.

It is found that mixtures 12 and 3 with 30 % replacement of FA exhibited the lowest sorptivity and WA capacity, respectively. This observation is related to CS and hardened density. It appears that under ambient curing, the low degree of reactivity of FA resulted in a more porous structure and increased WA capacity [77]. To achieve minimum WA, the FA-GGBFS blended GP concrete needs to have a binder concentration of 480 kg/m^3^, a GGBFS replacement percentage of 30 %, an AA to Binder ratio of 0.30, a Na_2_SiO_3_ to NaOH ratio of 2.0, an SH solution molarity of 12 M, and ambient curing conditions. On the other hand, mix 16 achieved the maximum absorption of 7.22 % because of its mix proportions, which had a reasonably high AAS/Binder ratio of 0.40. To achieve superior mechanical and durability performance, It seems that the pore shape that hampered the ease of water percolation was refined by a relatively low AA to Binder ratio. It's important to note that when FA completely replaced the GGBFS, the barrier to WA significantly decreased [78,79]. Fig. 14(a) illustrates typical plots of the WA and sorptivity for SC mixtures proportioned with various impacting variables. On the other hand, combinations made with lower AAS/Binder and higher NaOH solution molarity of 12 M were shown to have the maximum WA.

Biricik's [33] results, on the other hand, indicate a clear variation in the coefficient of capillarity depending on the curing conditions and Blaine's fineness. Specifically, the change in the capillarity coefficient was observed to be influenced by the Blaine fineness, with reduced fineness leading to an increased capillarity coefficient in GPMs. This suggests that curing conditions play an independent role in the value of the capillarity coefficient. While our study focused on WA and sorptivity, Biricik's study delved into the intricacies of the coefficient of capillarity and its dependence on factors such as curing conditions and Blaine fineness. These findings provide complementary insights into the WA behavior of geopolymeric materials.

A relationship between WA and sorptivity was developed using linear regression analysis in Eq. (17) and presented in Fig. 14(b). With a high correlation coefficient (R^2^ = 0.9877), it is possible to predict Sorptivity from WA or vice versa.

(17) $$Sorptivity=2.1693{WA}^{0.7291}$$

#### 3.1.8 Mix optimization by SNR

To find the ideal values of variables for SC, the SNR was employed. In accordance with the “larger is better” or “smaller is better” tenet, the blend with the greatest SNR was deemed to be the ideal design. For mechanical properties and slump of the SC, 540 kg/m^3^ binder content and molarity of NaOH of 16 M resulted in better results than the other levels of binder content and molarity of NaOH. From the SNR, AA to binder ratio of 0.35 shows better mechanical properties but for mechanical properties such as WA and sorptivity, AA to binder ratio of 0.3 resulted in better results. GGBFS of 30 % shows optimal replacement level for both mechanical as well as durability properties. AA ratio of 1.5 shows better mechanical properties whereas AA ratio of 2 shows better durability properties with the addition of GGBFS in the SC.

**Fig. 15:**
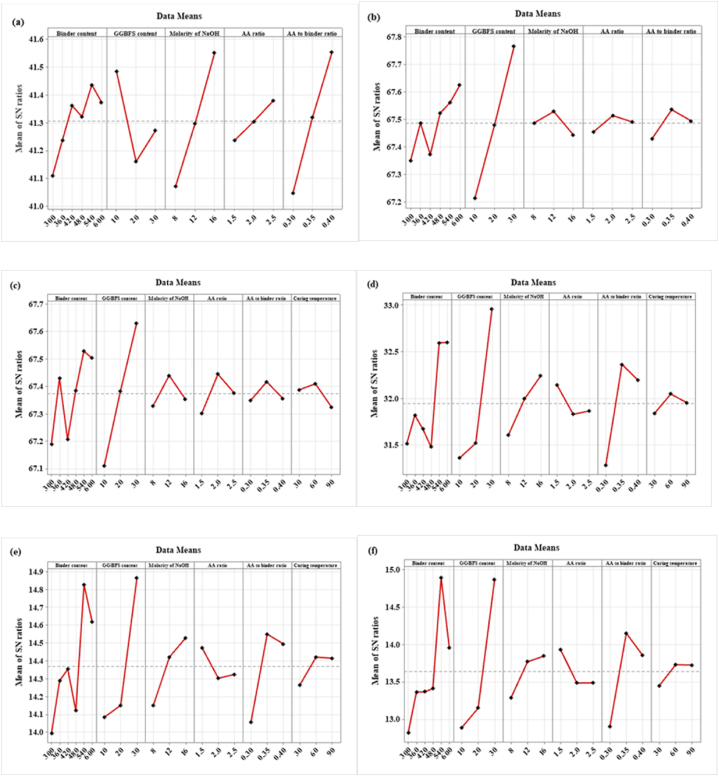

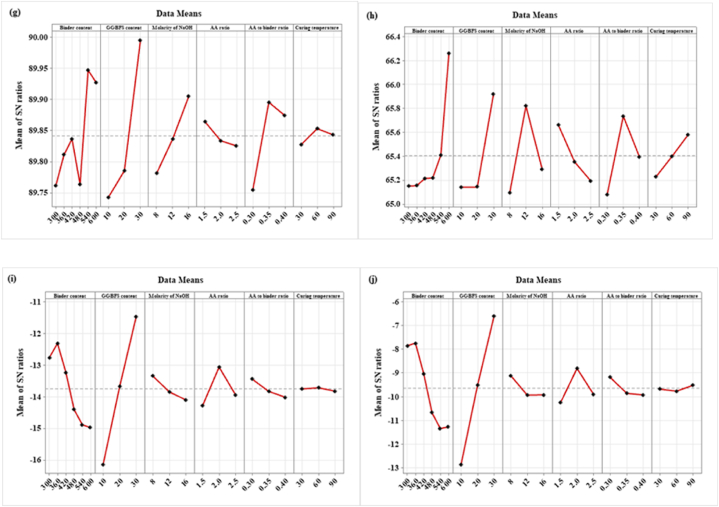
SNR graph for **(a)** Slump; **(b)** FD; **(c)** DD; **(d)** CS; **(e)** FS; **(f)** TS; **(g)** MOE; **(h)** IE; **(i)** WA; **(j)** Sorptivity.

Fig. 15 (a – j) shows the major effect graph of mean responses and SNR for all curing conditions. Furthermore, the contour plot and 3D surface plot of the considered parameters on the fresh, hardened, and durability properties of SC are also studied. These graphs are based on parameters such as binder content, GGBFS content, molarity of NaOH, AA ratio, AA to binder ratio, and CT.

#### 3.1.9 Optimization of the mix by contour plot

**Fig. 16:**
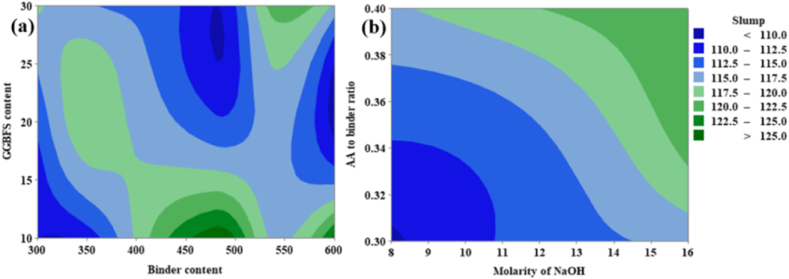
(a) Contour Plot of Slump v/s GGBFS content, Binder content; **(b)** Contour Plot of Slump v/s AA to binder ratio, Molarity of NaOH.

Contour plots were developed to understand the variations of the responses with respect to the influencing parameters and their levels [80]. From Fig. 16(a), it can be noted that the slump is inversely proportional to the GGBFS content and directly proportional to the binder content. From Fig. 16(a) it is evident that the maximum slump corresponding to GGBFS content varies from 10 % to 15 % and binder content varies from 450 kg/m^3^ to 500 kg/m^3^. From Fig. 16(b), it can be studied that the slump is directly proportional to the AA to binder ratio as well as the concentration of NaOH solution. From Fig. 16(b) maximum slump corresponding to AA to binder ratio varies from 0.38 to 0.40 and the concentration of NaOH solution varies from 15 M to 16 M.

**Fig. 17:**
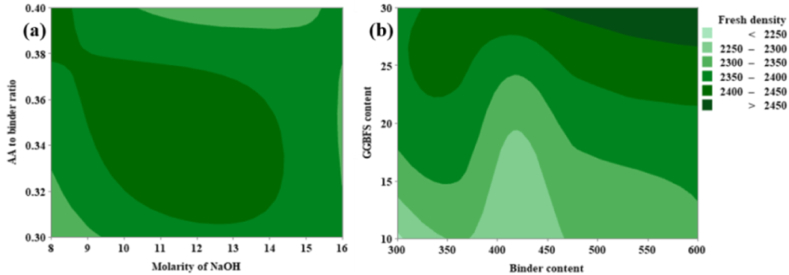
(a) Contour Plot of FD v/s GGBFS content, Binder content; **(b)** Contour Plot of FD v/s AA to binder ratio, Molarity of NaOH.

From Fig. 17(a), it can be noted that the FD is inversely proportional to the molarity of NaOH and directly proportional to the AA-to-binder ratio. From Fig. 17(a) it was possible to achieve maximum FD corresponding to AA to binder ratio varies from 0.38 to 0.40 and the concentration of NaOH solution varies from 8 M to 14 M. From Fig. 17(b) it is clear that the FD is directly proportional to both GGBFS content as well as binder content. From Fig. 17(b) it is clear that the maximum FD corresponding to GGBFS content varies from 25 % to 30 % and the binder content varies from 500 kg/m^3^ to 600 kg/m^3^.

**Fig. 18:**
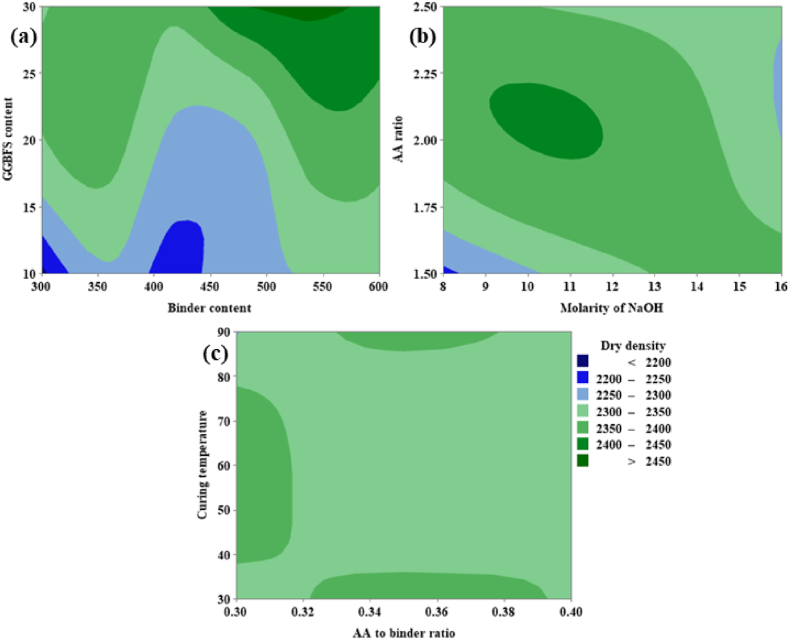
(a) Contour Plot of DD v/s GGBFS content, Binder content; **(b)** Contour plot of DD v/s AA ratio, Molarity of NaOH; **(c)** Contour Plot of DD v/s CT, AA to binder ratio.

From Fig. 18(a), it can be noted that the DD is directly proportional to both GGBFS and binder content. From Fig. 18(a) it is clear that the maximum DD corresponding to GGBFS content varies from 28 % to 30 % and the binder content varies from 490 kg/m^3^ to 550 kg/m^3^. From Fig. 18(b) it is clear that the DD was increased up to a particular level; beyond the optimum level, the value of DD gets reduced. The optimum range for both the parameters such as AA ratio and molarity of NaOH was 2.0–2.25 and 10 M − 12 M respectively. Similarly, from Fig. 18(c), it was clear that the optimum range for CT and AA to binder ratio was 40 °C–80 °C and 0.32–0.39 respectively.

**Fig. 19:**
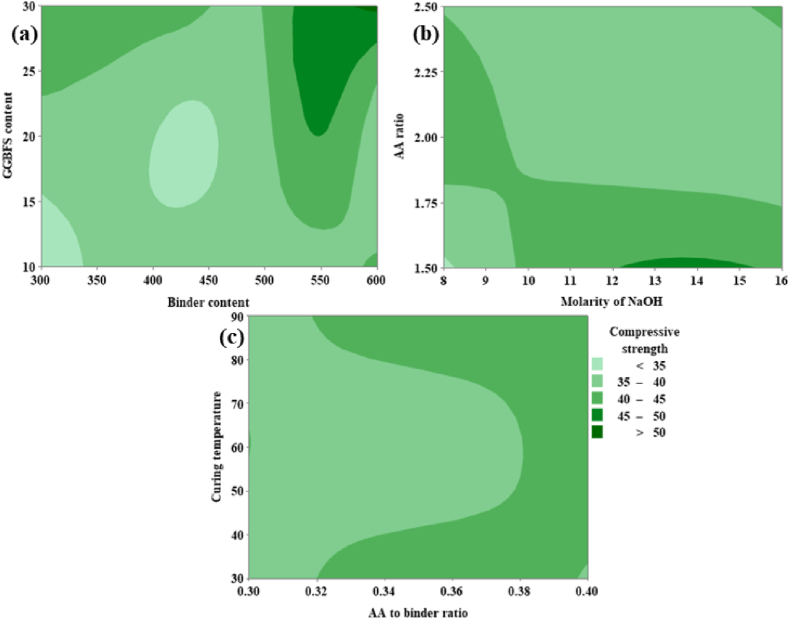
(a) Contour Plot of CS v/s GGBFS content, Binder content; **(b)** Contour Plot of CS v/s AA ratio, Molarity of NaOH; **(c)** Contour Plot of CS v/s CT, AA to binder ratio.

From Fig. 19(a), it can be noted that the CS is directly proportional to both the GGBFS content as well as binder content. From Fig. 19(a) it was possible to achieve maximum CS corresponding to GGBFs varies from 20 % to 30 % and the binder content varies from 520 kg/m^3^ to 600 kg/m^3^. From Fig. 19(b) it is shown that the CS is inversely proportional to the AA ratio and an optimum ranges from 1.75 to 2.50. For the molarity of NaOH optimum range is shown between 12 M and 15 M. From Fig. 19(c), CS shows a maximum value at CT in the range of 40 °C and 80 °C, and the AA to binder ratio shows directly proportional behavior between the range 0.32 and 0.40.

**Fig. 20:**
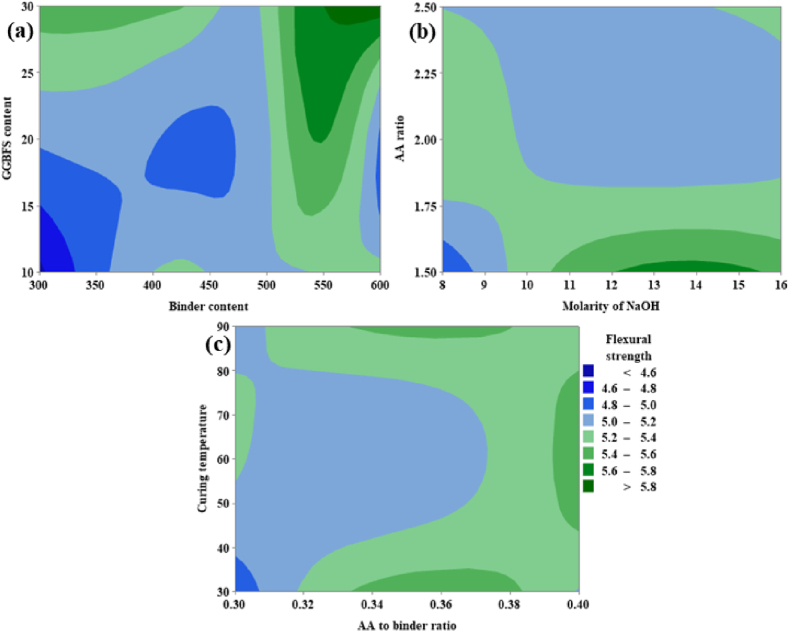
(a) Contour Plot of FS v/s GGBFS content, Binder content; **(b)** Contour Plot of FS v/s AA ratio, Molarity of NaOH; **(c)** Contour Plot of FS v/s CT, AA to binder ratio.

From Fig. 20(a), it can be noted that the FS is directly proportional to both the GGBFS content as well as binder content. From Fig. 20(a), it was possible to achieve maximum FS corresponding to GGBFS varies from 28 % to 30 % and the binder content varies from 550 kg/m^3^ to 600 kg/m^3^. From Fig. 20(b) it is clear that FS is inversely proportional to the AA ratio, and an optimum ranges from 1.75 to 2.50. For the molarity of NaOH optimum range is shown between 12 M and 15 M. From Fig. 20(c), FS shows a maximum value at CT in the range 55 °C and 80 °C, and the AA to binder ratio shows directly proportional behavior between the range 0.33–0.38.

**Fig. 21:**
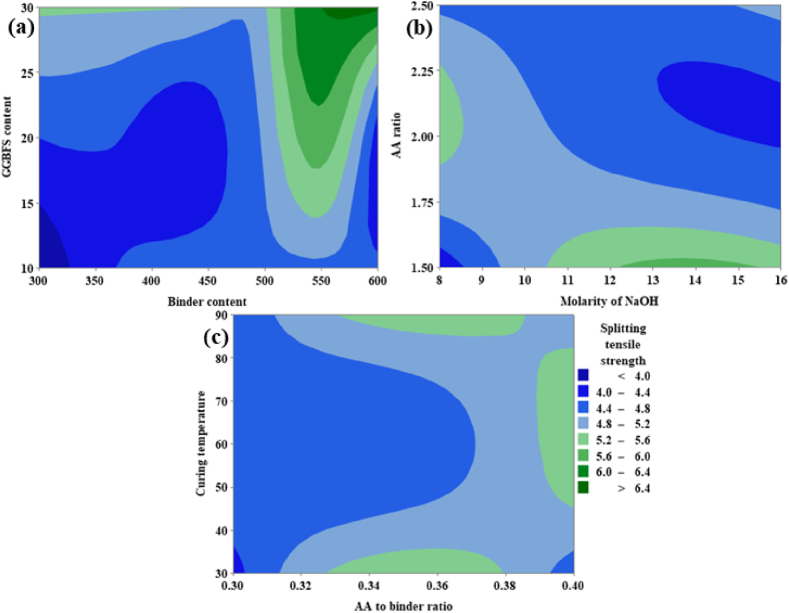
(a) Contour Plot of TS v/s GGBFS content, Binder content; **(b)** Contour Plot of TS v/s AA ratio, Molarity of NaOH; **(c)** Contour Plot of TS v/s CT, AA to binder ratio.

From Fig. 21(a), it can be noted that the TS is directly proportional to both the GGBFS content as well as binder content. From Fig. 21(a) it was possible to achieve maximum TS corresponding to GGBFS varies from 29 % to 30 % and the binder content varies from 550 kg/m^3^ to 600 kg/m^3^. From Fig. 21(b) it is clear that TS is inversely proportional to the AA ratio, and an optimum ranges from 1.80 to 2.25. For the molarity of NaOH optimum range shows between 12 M and 15.5 M. From Fig. 21(c), TS shows a maximum value at CT in the range of 55 °C and 80 °C, and the AA to binder ratio shows a direct proportional behavior between the range 0.33 and 0.38.

**Fig. 22:**
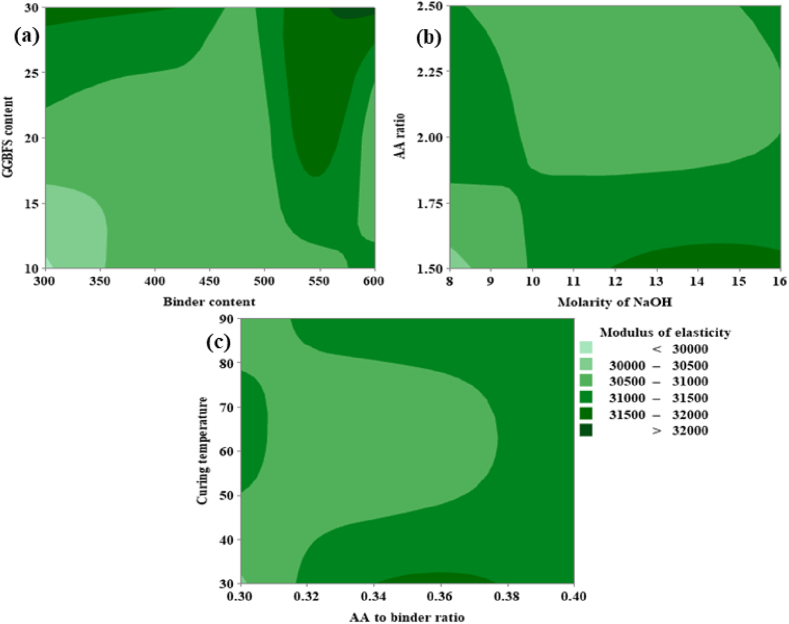
(a) Contour Plot of MOE v/s GGBFS content, Binder content; **(b)** Contour Plot of MOE v/s AA ratio, Molarity of NaOH; **(c)** Contour Plot of MOE v/s CT, AA to binder ratio.

From Fig. 22(a), it can be noted that the MOE is directly proportional to both the GGBFS content as well as binder content. From Fig. 22(a), it was possible to achieve a maximum MOE corresponding to GGBFS varies from 29 % to 30 % and the binder content varies from 560 kg/m^3^ to 600 kg/m^3^. From Fig. 22(b) it is evident that MOE is directly proportional to the AA ratio, and an optimum ranges from 1.75 to 2.25. For the molarity of NaOH optimum range shows between 12 M and 16 M. From Fig. 22(c), the MOE shows a maximum value at CT in the range of 50 °C and 80 °C, and the AA to binder ratio shows a directly proportional behavior between the range 0.34 and 0.37.

**Fig. 23:**
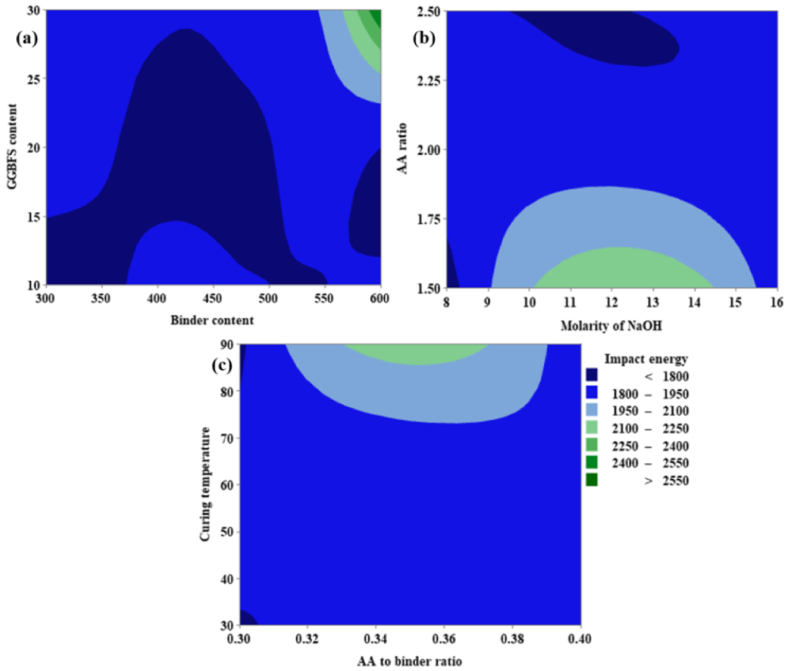
(a) Contour Plot of IE v/s GGBFS content, Binder content; **(b)** Contour Plot of IE v/s AA ratio, Molarity of NaOH; **(c)** Contour Plot of IE v/s CT, AA to binder ratio.

From Fig. 23(a), it can be noted that the IE is directly proportional to both the GGBFS content as well as binder content. From Fig. 23(a) it was possible to achieve maximum IE corresponding to GGBFS varies from 28 % to 30 % and the binder content varies from 580 kg/m^3^ to 600 kg/m^3^. From Fig. 23(b) it is evident that IE is directly proportional to the AA ratio, and an optimum ranges from 1.50 to 1.60. For the molarity of NaOH, the optimum range shows between 10 M and 14.5 M. From Fig. 23(c)–IE shows a maximum value at CT in the range of 85 °C and 90 °C, and the AA to binder ratio shows an optimum value between the range of 0.34 and 0.38.

**Fig. 24:**
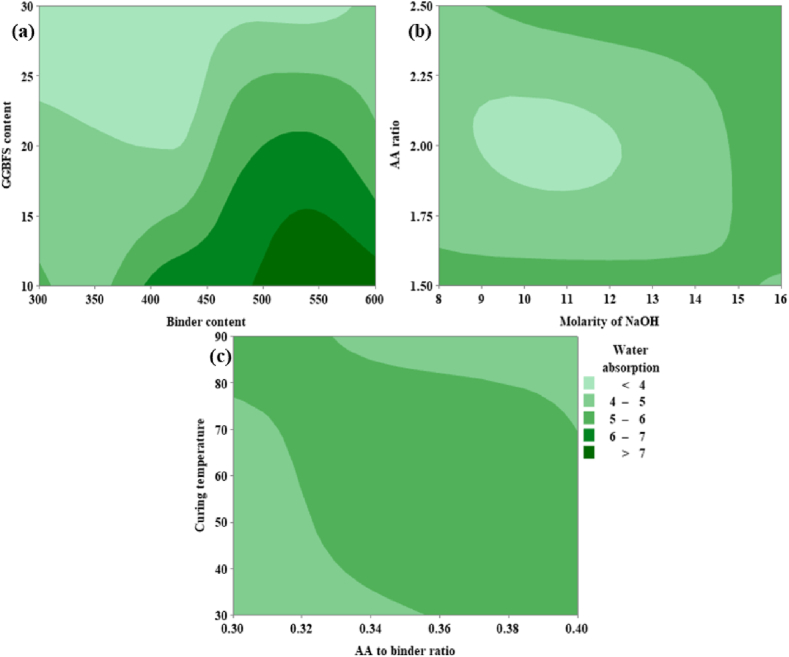
(a) Contour Plot of WA v/s GGBFS content, Binder content; **(b)** Contour Plot of WA v/s AA ratio, Molarity of NaOH; **(c)** Contour Plot of WA v/s CT, AA to binder ratio.

From Fig. 24(a), it can be noted that the WA is directly proportional to both the GGBFS content as well as binder content. From Fig. 24(a), it was possible to achieve a low WA corresponding to GGBFS varies from 23.5 % to 30 % and the binder content varies from 300 kg/m^3^ to 450 kg/m^3^. From Fig. 24(b) it is evident that WA is minimal to an extent; beyond the limit again the WA gets increased for both the parameters (i.e., AA ratio and molarity of NaOH). From Fig. 24(c), WA shows a minimum value at CT in the range of 30 °C and 75 °C, and the AA to binder ratio shows an optimum value for minimum WA in the range of 0.30 and 0.36.

**Fig. 25:**
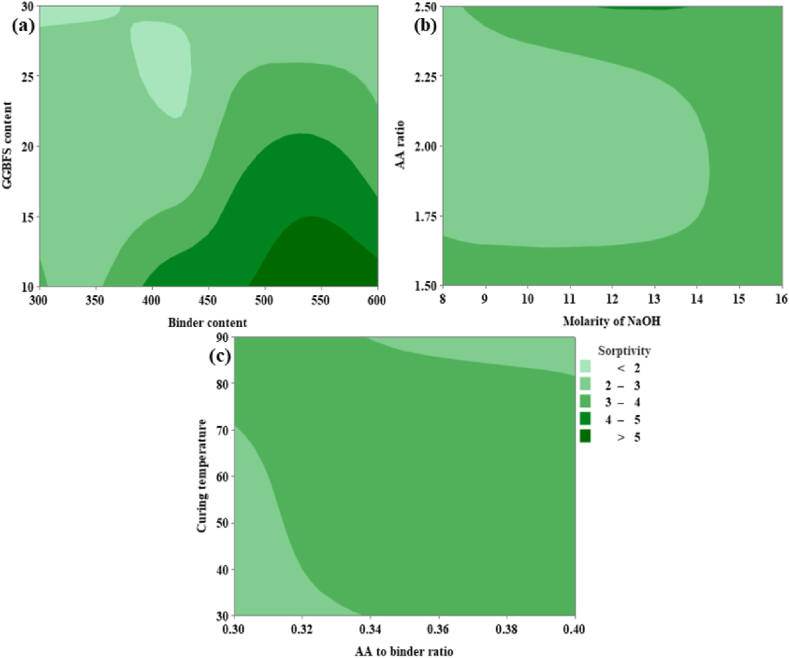
(a) Contour Plot of Sorptivity v/s GGBFS content, Binder content; **(b)** Contour Plot of Sorptivity v/s AA ratio, Molarity of NaOH; **(c)** Contour Plot of Sorptivity v/s CT, AA to binder ratio.

From Fig. 25(a), it can be noted that the sorptivity is inversely proportional to the GGBFS content whereas directly proportional to the binder content. From Fig. 25(a) it was possible to achieve minimum sorptivity corresponding to GGBFS that varies from 28 % to 30 % and the binder content varies from 310 kg/m^3^ to 350 kg/m^3^. From Fig. 25(b) it is evident that sorptivity is minimum at an AA ratio range of 1.75 and 2.50 and for optimum molarity range from 8 M to 14 M. From Fig. 25(c), sorptivity shows a minimum value at CT in the range of 30 °C and 70 °C and the AA to binder ratio shows an optimum value for minimum sorptivity in the range of 0.30 and 0.34.

#### 3.1.10 Optimization of the mix by 3D surface plot

**Fig. 26:**
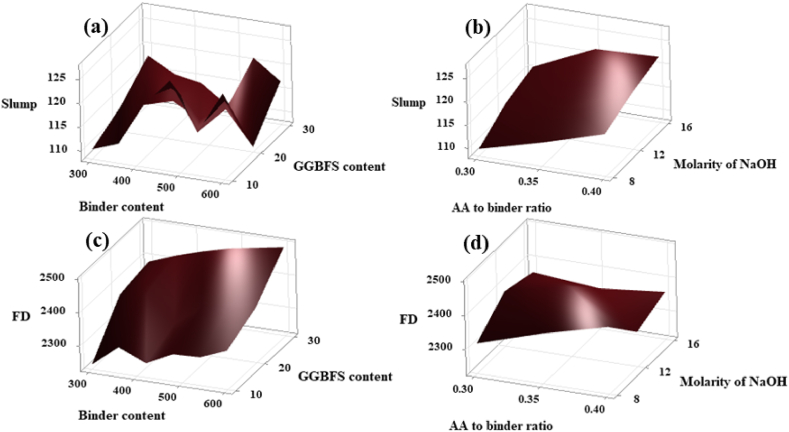
Surface Plot of **(a)** Slump v/s GGBFS content, Binder content; **(b)** Slump v/s Molar concentration of NaOH, AA/binder ratio; **(c)** FD v/s GGBFS content, Binder content; **(d)** FD v/s Molarity of NaOH, AA to binder ratio.

Fig. 26(a) and (b) represent the influence of individual parameters and their levels on the slump value of the SC [53]. It can be seen from Fig. 26(a) and (b) that the maximum slump value can be achieved with the binder content of 600 kg/m^3^, 10 wt% of GGBFS content, Molarity of NaOH solution of 16 M, and the AA to binder ratio of 0.4. Again, from Fig. 26(c) and (d) quantity of precursor of 600 kg/m^3^, GGBFS content of 30 wt%, the molarity of NaOH of 12 M, AA ratio of 1.5, and AA to binder ratio of 0.35 shows a higher FD.

**Fig. 27:**
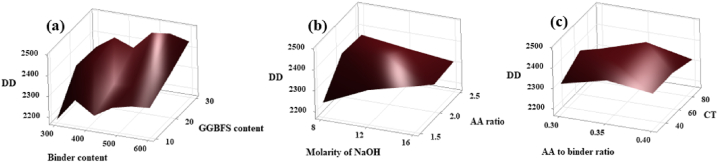
Surface Plot of **(a)** DD v/s GGBFS content, Binder content; **(b)** DD v/s AA ratio, Molarity of NaOH; **(c)** DD v/s CT, AA to binder ratio.

**Fig. 28:**
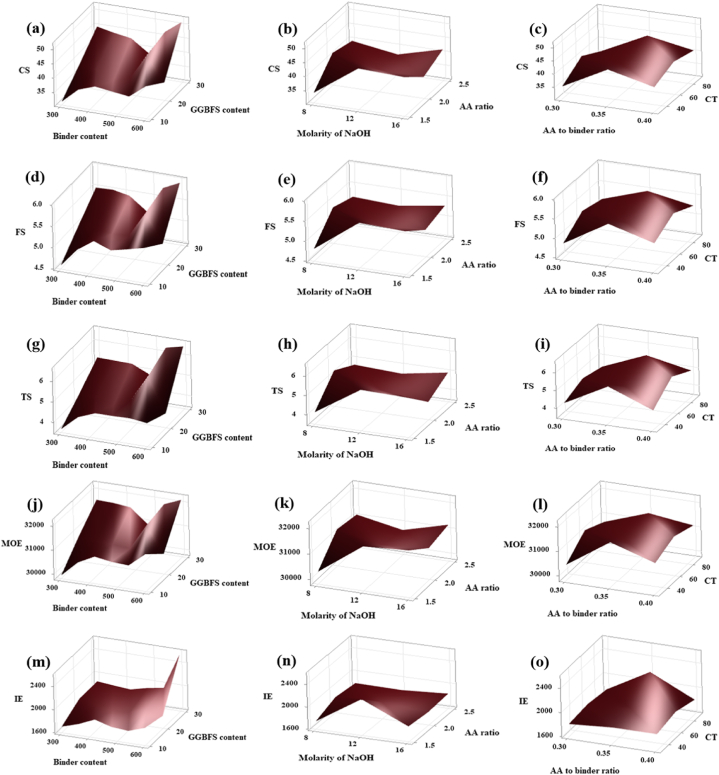
Surface Plot of **(a)** CS v/s GGBFS content, Binder content; **(b)** CS v/s AA ratio, Molarity of NaOH; **(c)** CS v/s CT, AA to binder ratio; **(d)** FS v/s GGBFS content, Binder content; **(e)** FS v/s AA ratio, Molarity of NaOH; **(f)** FS v/s CT, AA to binder ratio; **(g)** TS v/s GGBFS content, Binder content; **(h)** TS v/s AA ratio, Molarity of NaOH; **(i)** TS v/s CT, AA to binder ratio; **(j)** MOE v/s GGBFS content, Binder content; **(k)** MOE v/s AA ratio, Molarity of NaOH; **(l)** MOE v/s CT, AA to binder ratio; **(m)** IE v/s GGBFS content, Binder content; **(n)** IE v/s AA ratio, Molarity of NaOH; **(o)** IE v/s CT, AA to binder ratio.

Maximum DD value was obtained corresponding to their dependency levels shown in Fig. 27(a), (b), and (c). From Fig. 27 (a – c) it is clear that the maximum DD value was obtained for the quantity of precursor of 540 kg/m^3^, GGBFS content of 30 wt%, molarity of NaOH of 8 M, AA ratio of 2, AA/binder ratio of 0.4, and the CT of 60 °C. The same pattern was seen from the surface plot for the other mechanical properties such as CS, FS, TS, MOE, and IE which is shown in Fig. 28(a–o).

**Fig. 29:**
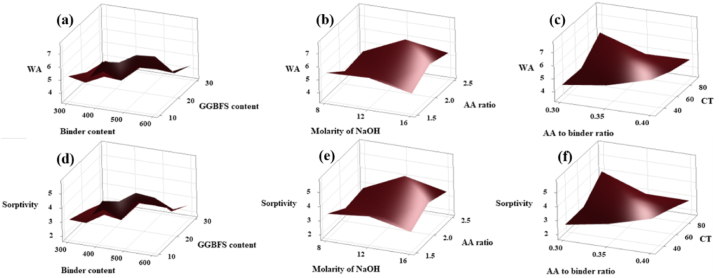
Surface Plot of **(a)** WA v/s GGBFS content, Binder content; **(b)** WA v/s AA ratio, Molarity of NaOH; **(c)** WA v/s CT, AA to binder ratio; **(d)** Sorptivity v/s GGBFS content, Binder content; **(e)** Sorptivity v/s AA ratio, Molarity of NaOH; **(f)** Sorptivity v/s CT, AA to binder ratio.

The minimum WA value was obtained from the surface plot which is shown in Fig. 29(a–c) for the quantity of precursor of 360 kg/m^3^, GGBFS content of 30 wt%, molarity of NaOH of 16 M, AA ratio of 1.5, AA to binder ratio of 0.3 and the CT of 60 °C. The minimum sorptivity value was obtained from the surface plot which is shown in Fig. 29(d–f) for the quantity of precursor of 300 kg/m^3^, GGBFS content of 30 wt%, molarity of NaOH of 16 M, AA ratio of 2.5, AA/binder ratio of 0.4 and the CT of 90 °C.

### 3.2 ANOVA

To determine how each component affected the characteristics of SC, ANOVA was carried out. Table 6 demonstrates that, with contributions of 35.076 % and 31.756 %, of AA to Binder and molarity of NaOH respectively which were the two parameters that had the greatest impact on the slump. Conversely, the AA to binder ratio and CT were the least impactful factors on DD, WA, and sorptivity with approximately 2 % contributions. It can be noted that, ANOVA results that the GGBFS content is the most influencing parameter for all the fresh as well as hardened properties of SC. Followed by binder content, molarity of NaOH, and AA ratio are the other influencing parameters on the different properties of SC. ANOVA results highlight that GGBFS replacement percentage, an increase in the quantity of precursor, and NaOH solution molarity led to an elevated mechanical strength such as CS, FS, TS, MOE, and IE, due to the presence of more CaO providing soluble Ca^2+^ ions, thereby improving the GGBFS's strength, high specific area, and granular structure as well as the hydroxide ions' rate of dissolution [52]. Conversely, increased Na_2_SiO_3_ to NaOH ratios decreased the mechanical strength due to the dense particle packing density, increased void content, and high silica content [81].

**Table 6:** ANOVA results.

Parameters	Contribution of parameters to the properties of SC (%)									
	Slump	FD	DD	CS	FS	STS	MOE	IE	WA	Sorptivity
Binder content	11.326	14.9	24.92	21.09	27.59	26.6	21.797	29.66	20.91	23.79
GGBFS content	16.416	78.72	63.67	48.51	42.9	48.02	49.93	25.70	69.06	66.98
Molarity of NaOH	31.756	2.09	3.26	6.53	8.85	3.78	10.62	18.21	2.39	2.16
AA ratio	5.426	1.16	4.90	1.9	2	2.76	1.23	7.68	5.49	4.5
AA to binder ratio	35.076	3.12	1.36	21.22	16.83	17.75	15.91	14.15	1.595	1.8
Curing temperature			1.90	0.74	1.83	1.10	0.54	4.59	0.565	0.77
Residual Error	–	–	–	–	–	–	–	–	–	–
Total	100.00	100.00	100.00	100.00	100.00	100.00	100.00	100.00	100.00	100.00

From the contribution table, it is found that CT is one of the least influencing parameters on the properties of SC. Table 7 indicates the optimal values of the parameters for the desired output.

**Table 7:** Optimization of parameters from ANOVA.

Parameters	Optimum levels									
	Slump	FD	DD	CS	FS	STS	MOE	IE	WA	Sorptivity
Binder content (kg/m^3^)	540	600	540	540	540	540	540	600	360	360
GGBFS content (%)	10	30	30	30	30	30	30	30	30	30
Molarity of NaOH (M)	16	12	12	16	16	16	16	12	8	8
AA ratio	2.5	2	2	1.5	1.5	1.5	1.5	1.5	2	2
AA to binder ratio	0.4	0.35	0.35	0.35	0.35	0.35	0.35	0.35	0.3	0.3
Curing temperature (^o^C)			60	60	60	60	60	90	60	90

### 3.3 Grey relation analysis

Data pre-processing is done to change the original sequence into a comparable sequence. The range of normalized numerical data is 0–1 [54]. Many different data pre-processing strategies are available depending on the data sequence's properties. In this study, the normalized value of the original sequence for CS, FD, DD, slump, TS, FS, MOE, and IE, which are larger-the-better, and for WA and sorptivity smaller-the-better performance characteristics can be expressed. The normalized values of CS, FD, DD, slump, TS, FS, MOE, IE, WA, and sorptivity are given in Table 8.

**Table 8:** Normalized experimental results.

Normalization									
CS	FD	DD	Slump	TS	FS	MOE	IE	Sorptivity	WA
1.0000	1.0000	1.0000	0.9444	1.0000	1.0000	1.0000	1.0000	0.3177	0.5249
0.6702	0.4841	0.4130	0.7778	0.7268	0.6903	0.8466	0.8602	0.1745	0.4513
0.3187	0.4127	0.4420	0.5000	0.4275	0.3935	0.5024	0.7844	0.0000	0.1734
0.7397	0.7460	0.6123	0.8333	0.7494	0.7161	0.9278	0.8981	0.3047	0.7055
0.7049	0.4206	0.3261	0.4444	0.7133	0.6710	0.6547	0.8673	0.1328	0.3064
0.3817	0.2937	0.2246	0.6667	0.4329	0.3806	0.4591	0.8152	0.0208	0.2090
0.6365	0.8889	0.8297	0.3333	0.6357	0.5419	0.8524	0.8602	0.7552	0.8052
0.8570	0.7381	0.6304	0.6111	0.8156	0.7226	0.5277	0.9313	0.0833	0.3872
0.4295	0.1944	0.3514	0.7222	0.4385	0.3032	0.4138	0.8436	0.0651	0.0238
0.6777	0.7341	0.6377	0.0000	0.6335	0.4645	0.8018	0.8555	0.7969	0.7791
0.7508	0.4921	0.6123	0.7778	0.6805	0.5161	0.3611	0.9123	0.4792	0.4109
0.6420	0.1111	0.0362	0.9444	0.5711	0.4129	0.1803	0.8697	0.0703	0.0000
0.7170	0.7222	0.5652	0.5556	0.6246	0.4645	0.4001	0.8815	1.0000	0.9881
0.3243	0.4325	0.3043	0.6111	0.2458	0.1097	0.2383	0.8009	0.5911	0.5487
0.2226	0.0556	0.0000	0.3333	0.0695	0.0000	0.0000	0.6066	0.0547	0.1378
0.4879	0.5952	0.5362	0.0556	0.6604	0.5677	0.2040	0.8602	0.9557	1.0000
0.7427	0.4087	0.3261	1.0000	0.8151	0.7419	0.1402	0.8720	0.3854	0.4014
0.0000	0.0000	0.0833	0.5556	0.0000	0.1032	0.0648	0.0000	0.1875	0.0855

The deviation sequences of CS, FD, DD, slump, TS, FS, MOE, impact, WA, and sorptivity are shown in Table 9. Next, the range between x_ij_ and x_0j_ is calculated using the grey relational coefficient. The closer x_ij_ and x_0j_ are, the higher the grey relationship coefficient.

**Table 9:** Deviation sequences.

Deviation sequence									
CS	FD	DD	Slump	TS	FS	MOE	IE	Sorptivity	WA
0.0000	0.0000	0.0000	0.0556	0.0000	0.0000	0.0000	0.0000	0.6823	0.4751
0.3298	0.5159	0.5870	0.2222	0.2732	0.3097	0.1534	0.1398	0.8255	0.5487
0.6813	0.5873	0.5580	0.5000	0.5725	0.6065	0.4976	0.2156	1.0000	0.8266
0.2603	0.2540	0.3877	0.1667	0.2506	0.2839	0.0722	0.1019	0.6953	0.2945
0.2951	0.5794	0.6739	0.5556	0.2867	0.3290	0.3453	0.1327	0.8672	0.6936
0.6183	0.7063	0.7754	0.3333	0.5671	0.6194	0.5409	0.1848	0.9792	0.7910
0.3635	0.1111	0.1703	0.6667	0.3643	0.4581	0.1476	0.1398	0.2448	0.1948
0.1430	0.2619	0.3696	0.3889	0.1844	0.2774	0.4723	0.0687	0.9167	0.6128
0.5705	0.8056	0.6486	0.2778	0.5615	0.6968	0.5862	0.1564	0.9349	0.9762
0.3223	0.2659	0.3623	1.0000	0.3665	0.5355	0.1982	0.1445	0.2031	0.2209
0.2492	0.5079	0.3877	0.2222	0.3195	0.4839	0.6389	0.0877	0.5208	0.5891
0.3580	0.8889	0.9638	0.0556	0.4289	0.5871	0.8197	0.1303	0.9297	1.0000
0.2830	0.2778	0.4348	0.4444	0.3754	0.5355	0.5999	0.1185	0.0000	0.0119
0.6757	0.5675	0.6957	0.3889	0.7542	0.8903	0.7617	0.1991	0.4089	0.4513
0.7774	0.9444	1.0000	0.6667	0.9305	1.0000	1.0000	0.3934	0.9453	0.8622
0.5121	0.4048	0.4638	0.9444	0.3396	0.4323	0.7960	0.1398	0.0443	0.0000
0.2573	0.5913	0.6739	0.0000	0.1849	0.2581	0.8598	0.1280	0.6146	0.5986
1.0000	1.0000	0.9167	0.4444	1.0000	0.8968	0.9352	1.0000	0.8125	0.9145

The grey relation grade shows the degree of similarity between the comparability sequence and the reference sequence. The optimal option would be the trial with the greatest grey relational grade with the reference sequence, which indicates that the comparability sequence is most comparable to the reference sequence. The grey relationship coefficients and grade for each trial are displayed in Table 10.

**Table 10:** Grey relationship coefficients and their grade.

Grey relation co-efficient - GRC										Grade
CS	FD	DD	Slump	TS	FS	MOE	IE	Sorptivity	WA	
1.0000	1.0000	1.0000	0.9000	1.0000	1.0000	1.0000	1.0000	0.4229	0.5128	0.8836
0.6025	0.4922	0.4600	0.6923	0.6467	0.6175	0.7652	0.7815	0.3772	0.4768	0.5912
0.4233	0.4599	0.4726	0.5000	0.4662	0.4519	0.5012	0.6987	0.3333	0.3769	0.4684
0.6576	0.6632	0.5633	0.7500	0.6662	0.6379	0.8738	0.8307	0.4183	0.6293	0.6690
0.6289	0.4632	0.4259	0.4737	0.6356	0.6031	0.5915	0.7903	0.3657	0.4189	0.5397
0.4471	0.4145	0.3920	0.6000	0.4686	0.4467	0.4804	0.7301	0.3380	0.3873	0.4705
0.5790	0.8182	0.7459	0.4286	0.5785	0.5219	0.7721	0.7815	0.6713	0.7197	0.6617
0.7776	0.6563	0.5750	0.5625	0.7306	0.6432	0.5142	0.8792	0.3529	0.4493	0.6141
0.4671	0.3830	0.4353	0.6429	0.4710	0.4178	0.4603	0.7617	0.3485	0.3387	0.4726
0.6081	0.6528	0.5798	0.3333	0.5770	0.4829	0.7161	0.7757	0.7111	0.6936	0.6131
0.6673	0.4961	0.5633	0.6923	0.6101	0.5082	0.4390	0.8508	0.4898	0.4591	0.5776
0.5827	0.3600	0.3416	0.9000	0.5383	0.4599	0.3789	0.7932	0.3497	0.3333	0.5038
0.6386	0.6429	0.5349	0.5294	0.5712	0.4829	0.4546	0.8084	1.0000	0.9768	0.6640
0.4253	0.4684	0.4182	0.5625	0.3987	0.3596	0.3963	0.7153	0.5501	0.5256	0.4820
0.3914	0.3462	0.3333	0.4286	0.3495	0.3333	0.3333	0.5597	0.3459	0.3670	0.3788
0.4940	0.5526	0.5188	0.3462	0.5955	0.5363	0.3858	0.7815	0.9187	1.0000	0.6129
0.6602	0.4582	0.4259	1.0000	0.7300	0.6596	0.3677	0.7962	0.4486	0.4551	0.6002
0.3333	0.3333	0.3529	0.5294	0.3333	0.3580	0.3484	0.3333	0.3810	0.3535	0.3656

For each level of controllable parameters, the means of the grey relational grade were calculated from Table 10 and compiled in Table 11. As a result, Table 11 provides the most and least impacting factors. From the GRA, it was observed that GGBFS inclusion as FA replacement was the most significant parameter followed by other variables such as binder content, molar concentration of NaOH, AA/binder ratio, AA ratio, and CT were the least significant parameters.

**Table 11:** Response table for grey relation grade.

Parameters/Levels	A	B	C	D	E	F	Rank
BC	0.648	0.560	0.583	0.565	0.508	0.526	2(0.139)
GGBFS	0.684	0.567	0.443				1(0.241)
NaOH	0.597	0.554	0.543				4(0.053)
AA ratio	0.573	0.562	0.560				6(0.014)
AA to B	0.623	0.532	0.540				3(0.090)
CT	0.582	0.553	0.560				5(0.030)
Average of Grade	0.565						

### 3.4 Prediction of regression equations

**Fig. 30:**
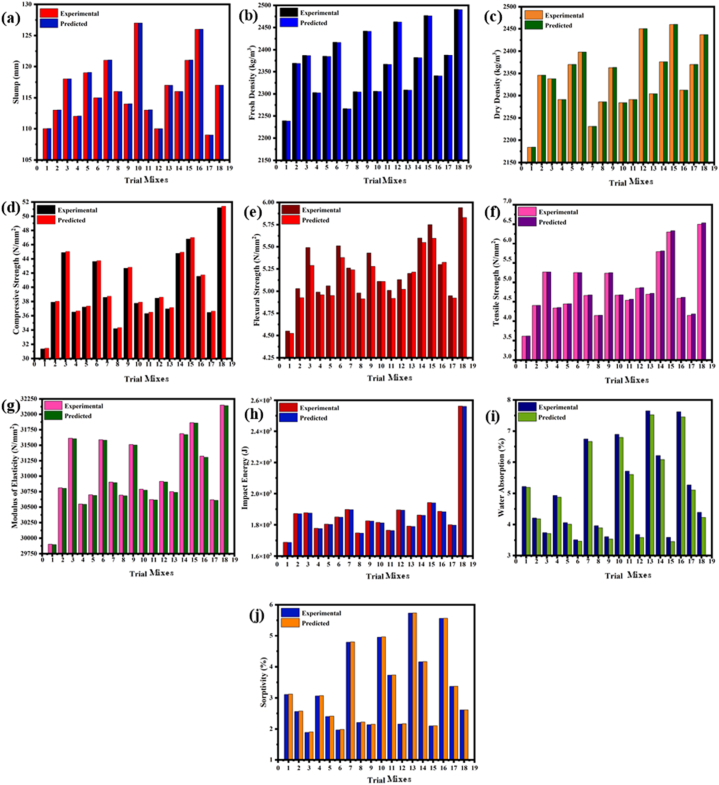
Experimental and predicted value graph for, **(a)** Slump; **(b)** FD; **(c)** DD; **(d)** CS; **(e)** FS; **(f)** TS; **(g)** MOE; **(h)** IE; **(i)** WA; **(j)** Sorptivity.(28)$RMSE=\sqrt{\frac{1}{n}}\sum_{i=1}^{n}{\left(Y_{i}-{\hat{Y}}_{i}\right)}^{2}$

Regression equations for the slump (y_1_), FD (y_2_), DD (y_3_), CS (y_4_), FS (y_5_), TS (y_6_), MOE (y_7_), IE (y_8_), WA (y_9_), and sorptivity (y_10_) were developed, based on the experimental findings from the study and more than 95 % confidence level is achieved. All the variable parameters were incorporated into the generated model i.e., binder content(x_1_), GGBFS content(x_2_), molarity of NaOH(x_3_), AA ratio(x_4_), AA to binder ratio(x_5_), and CT (x_6_). Fig. 30 (a – j) demonstrates a clear agreement between the output of the mathematical model and the outcomes of the experiment. The model's significance and the significance of all its components were tested at a confidence level of over 95 %. The regression models are presented below based on the findings that meet the required threshold of P < 0.05. i.e., Eq.(18), (19), (20), (21), (22), (23), (24), (25), (26), (27). Root Mean Square Error (RMSE) and Mean Absolute Error (MAE) were also calculated by using Eq. (28), (29), (30) for each parameter and tabulated in Table 12.

(18) $$y_{1}=82.02-0.3259x_{1}-3.702x_{2}+11.58x_{3}-48.62x_{4}+499.6x_{5}-0.000167x_{1}^{2}+0.004667x_{2}^{2}-0.3181x_{3}^{2}+11.02x_{4}^{2}-1653x_{5}^{2}+0.001704x_{1}x_{2}-0.01046x_{1}x_{3}+0.01630x_{1}x_{4}+1.630x_{1}x_{5}+0.1033x_{2}x_{3}+0.4933x_{2}x_{4}+0.8000x_{2}x_{5}$$

(19) $$y_{2}=1325-1.116x_{1}+22.13x_{2}-46.11x_{3}+49.25x_{4}+5808x_{5}+0.000407x_{1}^{2}-0.01700x_{2}^{2}-0.1885x_{3}^{2}-61.97x_{4}^{2}-3517x_{5}^{2}+0.004167x_{1}x_{2}+0.1053x_{1}x_{3}+0.7378x_{1}x_{4}-5.467x_{1}x_{5}+0.1483x_{2}x_{3}-6.213x_{2}x_{4}-3.733x_{2}x_{5}$$

(20) $$y_{3}=3540-1.014x_{1}-5.416x_{2}-144.1x_{3}+530.5x_{4}-8653x_{5}+18.24x_{6}-0.002111x_{1}^{2}-0.3878x_{2}^{2}-1.863x_{3}^{2}-89.92x_{4}^{2}+12312x_{5}^{2}-0.1482x_{6}^{2}-0.005306x_{1}x_{2}+0.3051x_{1}x_{3}+2.587x_{2}x_{3}-1.973x_{2}x_{4}+30.13x_{2}x_{5}$$

(21) $$y_{4}=902.4-0.6599x_{1}-2.645x_{2}-40.09x_{3}-109.8x_{4}-3181x_{5}+4.582x_{6}-0.000256x_{1}^{2}-0.06925x_{2}^{2}-0.1614x_{3}^{2}+26.95x_{4}^{2}+4563x_{5}^{2}-0.03460x_{6}^{2}-0.000752x_{1}x_{2}+0.08317x_{1}x_{3}+0.3640x_{2}x_{3}+1.305x_{2}x_{4}+3.275x_{2}x_{5}$$

(22) $$y_{5}=92.38-0.05670x_{1}-0.2201x_{2}-3.786x_{3}-10.35x_{4}-340.3x_{5}+0.4388x_{6}-0.000032x_{1}^{2}-0.007570x_{2}^{2}-0.01704x_{3}^{2}+2.525x_{4}^{2}+485.4x_{5}^{2}-0.003306x_{6}^{2}-0.000079x_{1}x_{2}+0.007975x_{1}x_{3}+0.03362x_{2}x_{3}+0.1289x_{2}x_{4}+0.3213x_{2}x_{5}$$

(23) $$y_{6}=104-0.06091x_{1}-0.5799x_{2}-4.806x_{3}-12.15x_{4}-377.1x_{5}+0.5777x_{6}-0.000053x_{1}^{2}-0.008115x_{2}^{2}-0.02809x_{3}^{2}+2.963x_{4}^{2}+524.2x_{5}^{2}-0.004394x_{6}^{2}-0.000084x_{1}x_{2}+0.01027x_{1}x_{3}+0.04787x_{2}x_{3}+0.1533x_{2}x_{4}+1.042x_{2}x_{5}$$

(24) $$y_{7}=165864-93.59x_{1}-276x_{2}-6016x_{3}-15781x_{4}-519883x_{5}+681x_{6}-0.04425x_{1}^{2}-11.93x_{2}^{2}-20.68x_{3}^{2}+3815x_{4}^{2}+743552x_{5}^{2}-5.128x_{6}^{2}-0.1562x_{1}x_{2}+12.45x_{1}x_{3}+50.89x_{2}x_{3}+206.8x_{2}x_{4}+409.7x_{2}x_{5}$$

(25) $$y_{8}=14008-18.54x_{1}-7.578x_{2}-337.2x_{3}-2602x_{4}-36214x_{5}+53.80x_{6}+0.005079x_{1}^{2}-0.3837x_{2}^{2}-7.447x_{3}^{2}+658.1x_{4}^{2}+57333x_{5}^{2}-0.3779x_{6}^{2}+0.1085x_{1}x_{2}+1.086x_{1}x_{3}+2.434x_{2}x_{3}+14.62x_{2}x_{4}-140.4x_{2}x_{5}$$

(26) $$y_{9}=-175.8+0.1359x_{1}-0.1488x_{2}+7.124x_{3}+9.633x_{4}+751.7x_{5}-0.6869x_{6}+0.000036x_{1}^{2}+0.01807x_{2}^{2}-0.01169x_{3}^{2}-2.793x_{4}^{2}-1100x_{5}^{2}+0.005117x_{6}^{2}-0.000731x_{1}x_{2}-0.01316x_{1}x_{3}-0.04943x_{2}x_{3}-0.1768x_{2}x_{4}+0.7093x_{2}x_{5}$$

(27) $$y_{10}=-133+0.1082x_{1}-0.1031x_{2}+5.359x_{3}+6.676x_{4}+555.2x_{5}-0.4777x_{6}+0.000019x_{1}^{2}+0.01388x_{2}^{2}-0.02055x_{3}^{2}-1.958x_{4}^{2}-816.6x_{5}^{2}+0.003539x_{6}^{2}-0.000738x_{1}x_{2}-0.009339x_{1}x_{3}-0.03632\text{bc}-0.1312x_{2}x_{4}+0.6707x_{2}x_{5}$$

**Table 12:** RMSE, MAE, and R^2^ values.

Properties	RMSE	MAE	R^2^
Slump (mm)	0.031187	0.024789	1.000
Fresh Density (kg/m^3^)	0.670926	0.665778	1.000
Dry Density (kg/m^3^)	0.239225	0.191111	1.000
Compressive strength (N/mm^2^)	0.156577	0.154078	0.999
Flexural strength (N/mm^2^)	0.097021	0.078892	0.911
Tensile strength (N/mm^2^)	0.019281	0.015597	0.999
Modulus of elasticity (N/mm^2^)	11.04863	10.27778	1.000
Impact energy (J)	2.055852	2.019686	1.000
Water absorption (%)	0.102176	0.09092	0.995
Sorptivity (%)	0.011221	0.0104	1.000

[y_1_ = slump; y_2_ = FD; y_3_ = DD; y_4_ = CS; y_5_ = FS; y_6_ = TS; y_7_ = MOE; y_8_ = IE; y_9_ = WA; y_10_ = sorptivity; x_1_ = Binder content; x_2_ = GGBFS content; x_3_ = Molarity of NaOH; x_4_ = AA ratio; x_5_ = AA/binder ratio; x_6_ = Curing temperature].Where, n - number of data points, Y_i_-actual value, and $\hat{Y_{i}}$ -predicted value.

(29) $$MAE=\frac{1}{n}\sum_{i=1}^{n}\left|Y_{i}-{\hat{Y}}_{i}\right|$$

(30) $$R^{2}=1-\frac{\sum_{i=1}^{n}{\left(Y_{i}-{\hat{Y}}_{i}\right)}^{2}}{\sum_{i=1}^{n}{\left(Y_{i}-\bar{Y}\right)}^{2}}$$

Where, $\bar{Y}$-mean of the actual values, Y_i_ -actual value, and $\hat{Y_{i}}$-predicted value.

### 3.5 Confirmation test

Conformation experiments must be carried out to validate the Taguchi estimated optimum conditions. The reaction at projected optimum conditions was estimated and verified using the predicted SNR (Ꜫ) [82], derived using Eq. (31).

(31) $$\varepsilon_{\text{predicted}}=\varepsilon_{l}+\sum_{i=1}^{x}\left(\varepsilon_{o}-\varepsilon_{l}\right)$$

The confirmation experiments were carried out with the Taguchi predicted optimum conditions for all the attributes in this work, and the outcomes are reported in Table 13. The predicted optimum conditions for both fresh and mechanical properties improve the performance characteristic results. According to Table 6, all of the SC's attributes have SNRs that are very near to those of the optimum condition. The SNR improvement found at the optimal condition for the slump, FD, DD, CS, FS, TS, MOE, IE, WA, and sorptivity were 0.8771, 0.594, 0.3867, 1.95, 1.075, 2.425, 0.416, 0.9075, 3.1742, 3.208 and 8.99 respectively when compared to the initial parameters shown in Table .13. It was discovered from the conformation experiments that the optimum conditions obtained by Taguchi method outperform the initial parameter conditions. From the Taguchi predicted optimum conditions percentage increase in SNR for the slump, FD, DD, CS, FS, TS, MOE, and IE were found to be 1.57 %, 0.76 %, 1.54 %, 3.22 %, 1.52 %, 6.15 %, 0.165 %, and 1.17 % respectively. Whereas, WA and sorptivity percentage reduction in SNR were 5.84 % and 8.99 % respectively.

**Table 13:** Confirmation test results.

	Initial parameters	Optimal parameters			Initial parameters	Optimal parameters	
		Prediction	Experiment			Prediction	Experiment
Level	x_1D_x_2A_x_3C_x_4C_x_5B_	x_1E_x_2A_x_3C_x_4C_x_5C_		Level	x_1F_x_2C_x_3B_x_4A_x_5B_x_6C_	x_1E_x_2C_x_3C_x_4A_x_5B_x_6B_	
Slump	127	–	129	TS	6.5	–	6.9
SNR	41.55	42.18	42.42	SNR	14.89	17.23	17.31
Improvement in SNR	0.87			Improvement in SNR	2.42		
Percentage Increase	1.57			Percentage Increase	6.15		
Level	x_1F_x_2C_x_3B_x_4A_x_5B_	x_1F_x_2C_x_3B_x_4B_x_5B_		Level	x_1F_x_2C_x_3B_x_4A_x_5B_x_6C_	x_1E_x_2C_x_3C_x_4A_x_5B_x_6B_	
FD	2491	–	2510	MOE	32150	–	32203
SNR	67.77	68.02	68.36	SNR	90	90.25	90.41
Improvement in SNR	0.59			Improvement in SNR	0.41		
Percentage Increase	0.76			Percentage Increase	0.16		
Level	x_1E_x_2C_x_3A_x_4B_x_5C_x_6B_	x_1E_x_2C_x_3B_x_4B_x_5B_x_6B_		Level	x_1F_x_2C_x_3B_x_4A_x_5B_x_6C_	x_1E_x_2C_x_3B_x_4A_x_5B_x_6C_	
DD	2460	–	2498	IE	2562	–	2592
SNR	67.63	68.00	68.01	SNR	66.26	67.11	67.16
Improvement in SNR	0.38			Improvement in SNR	0.90		
Percentage Increase	1.54			Percentage Increase	1.17		
Level	x_1F_x_2C_x_3B_x_4A_x_5B_x_6C_	x_1E_x_2C_x_3C_x_4A_x_5B_x_6B_		Level	x_1B_x_2C_x_3C_x_4A_x_5A_x_6B_	x_1F_x_2A_x_3C_x_4A_x_5C_x_6C_	
CS	51.22	–	52.87	WA	3.59	–	3.38
SNR	32.96	34.61	34.91	SNR	−16.14	−18.52	−19.31
Improvement in SNR	1.95			Improvement in SNR	3.17		
Percentage Increase	3.22			Percentage reduction	5.84		
Level	x_1F_x_2C_x_3B_x_4A_x_5B_x_6C_	x_1E_x_2C_x_3C_x_4A_x_5B_x_6B_		Level	x_1A_x_2C_x_3C_x_4C_x_5C_x_6C_	x_1E_x_2A_x_3B_x_4A_x_5C_x_6B_	
FS	5.94	–	6.03	Sorptivity	1.89	–	1.72
SNR	14.86	15.82	15.93	SNR	−12.85	−15.80	−16.06
Improvement in SNR	1.07			Improvement in SNR	3.20		
Percentage Increase	1.51			Percentage reduction	8.99		

[x_1_ = Binder content; x_2_ = GGBFS content; x_3_ = Molarity of NaOH; x_4_ = AA ratio; x_5_ = AA/binder ratio; x_6_ = Curing temperature and A, B, C, D, E, and F represent the levels of each influencing parameters given in Table 2].

## 4 Microstructural analysis

**Fig. 31:**
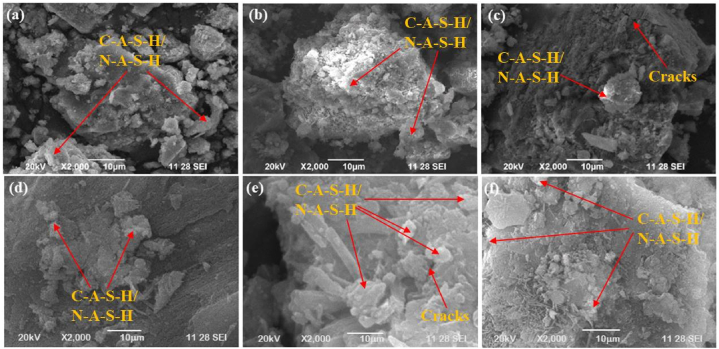
SEM micrographs of SC for **(a)** T_3_; **(b)** T_6_; **(c)** T_9_; **(d)** T_12_; **(e)** T_15_; **(f)** T_18_.

To examine SC's microstructure, eighteen distinct mixtures were created by changing the binder amount, GGBFS content, AA ratio, AA to FA ratio, concentration of SH, and CT. To verify their microstructural characteristics, both ambient curing and oven curing approaches were adopted on the developed SC samples. SEM is used to analyze the microstructural characteristics. Fig. 31(a–f) displays the SEM pictures of the SC samples after a 28-day curing period. In contrast to the mix with 10 % GGBFS, the SC mix with 30 % GGBFS (T_18_) exhibits a dense microstructure and fewer pores, as can be seen in Fig. 31(f). Mix 18 had spherical, angular, and fine microcracks as depicted in Fig. 31, and was composed of 30 % GGBFS and 70 % FA. Thus, the formation of C S H and C A S H gels resulted in the compaction of the microstructure of the GP concrete [81,83]. It is difficult to find the unreacted FA-GGBFS particles in mixtures containing 10, and 20 % GGBFS. Fig. 31(a) depicts a heterogeneous microstructure characterized by significant voids and unreacted GGBFS. Notably, mix 1 with 10 % GGBFS had the lowest CS value of all the SC mixes. Unreacted particles can be found when the GGBFS level of the mixture increases. When class F FA is dissolved, the polymeric gel is created with barely detectable amounts of calcium oxides, and the predominant byproduct is N A S H. C A S H gel is produced as a result of an increase in calcium intensity caused by an increase in the GGBFS concentration [81]. An increase in the degree of C A S H and N A S H gel integration was observed in the ideal mixture, as opposed to mixing 18 with 30 % GGBFS mixture. These intermixes led to the best cube CS of the mixes, which was 51.22 MPa. According to the above data, the inclusion of GGBFS results in an extra-binding product that alters the early stages of SC setting behavior.

## 5 Sustainability index

### 5.1 Cost efficiency

**Fig. 32:**
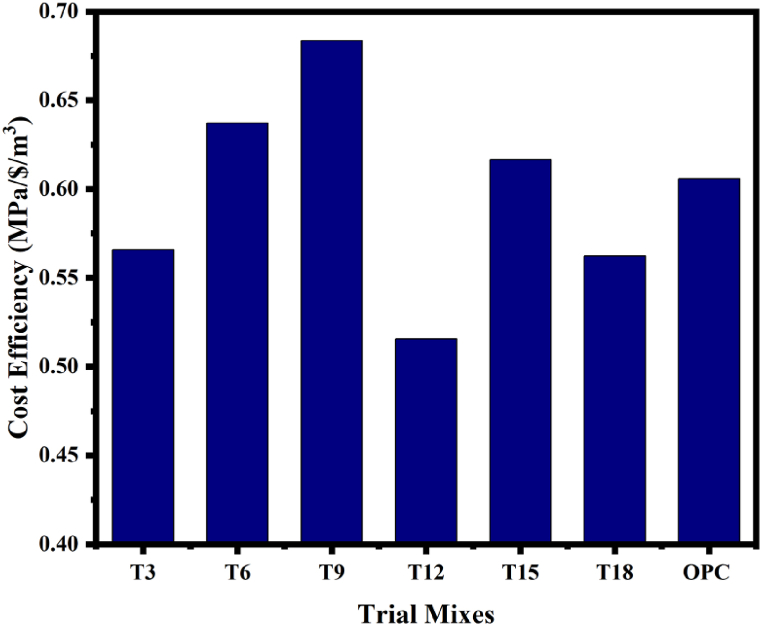
Cost efficiency of SC with OPC.

The ratio between strength and cost was assessed to classify the sustainability of FA GGBFS based SC in terms of its cost effectiveness [84]. The calculation of the SC cost was determined by assessing the prices of locally sourced ingredients. The material expenses include OPC at $130.5 per metric ton, FA at $13.05 per metric ton, GGBFS at $67.88 per metric ton, coarse aggregates at $9.14 per metric ton, fine aggregates at $7.31 per metric ton, NaOH at $411.18 per metric ton, and Na_2_SiO_3_ at $117.48 per metric ton. Because the raw materials are closer to the study location, the transportation costs for all the concrete mix components are not considered. Fig. 32 illustrates the cost efficiency of trail mixes. An improvement in cost efficiency translates into greater manufacturing of concrete effectiveness. According to Fig. 32, when compared to other mixes, the T_9_ mix offers the highest cost efficiency. The price of producing concrete rises as GGBFS content increases. On the other hand, a rise in the GGBFS proportion improves the SC mix's overall cost efficiency in terms of efficiency. Table 14 presents an assessment of the cost efficiency of concrete made with a blend of FA and GGBFS compared to OPC based concrete. The table illustrates that the production costs of FA GGBFS based concrete are comparatively lower than those of OPC concrete. Furthermore, the cost effectiveness of OPC based concrete is on par with that of SC containing 30 % GGBFS. So it is possible to suggest FA GGBFS mixed SC as an alternative to traditional cement-based concretes.

**Table 14:** Cost efficiency of SC and OPC concrete (for 1 m^3^).

Sl.No	Materials	Rate ($/MT)	T_3_		T_6_		T_9_		T_12_		T_15_		T_18_		OPC (IS-10262-2019)	
			quantity	cost	quantity	cost	quantity	cost	quantity	cost	quantity	coat	quantity	cost	quantity	cost
1	OPC	130.5	–	–	–	–	–	–	–	–	–	–	–	–	428	55.85
2	FA	13.05	210	2.7405	252	3.2886	294	3.8367	336	4.3848	378	4.9329	420	5.481	–	–
3	GGBFS	67.88	90	6.1092	108	7.331	126	8.5528	144	9.7747	162	10.9965	180	12.2184	–	–
4	SF	391.6	–	–	–	–	–	–	–	–	–	–	–	–	26.75	10.48
5	C.Agg	9.14	1264.564	11.5581	1334.957	12.2015	1306.53	11.9416	1220.863	11.1586	1183.256	10.8149	1095.429	10.0122	1219	11.14
6	F.Agg	7.31	655.7	4.7931	692.2	5.0599	677.46	4.9522	633.04	4.6275	613.54	4.4849	568	4.1521	589	4.31
7	NaOH	411.18	76.8	31.5786	69.12	28.4207	47.04	19.34	69.12	28.4207	69.12	28.4207	100.8	41.4469	–	–
8	SP	–	–	–	–	–	–	–	–	–	–	–	–	–	2.67	10.46
9	Na_2_SiO_3_	117.48	192	22.5562	103.68	12.1803	117.6	13.8156	138.24	16.24	138.24	16.2404	151.2	17.7629	–	–
10	Total cost ($/m^3^)			79.3357	–	68.482	–	62.4389	–	74.6063	–	75.8903	–	91.0735	–	93.28
11	28th day Compressive strength			44.89	–	43.64	–	42.69	–	38.47	–	46.8	–	51.22	–	56.5
12	Cost Efficiency (MPa/$/m^3^)			0.565823	–	0.637248	–	0.683708	–	0.51564	–	0.61668	–	0.562403	–	0.605703

### 5.2 Energy efficiency

**Fig. 33:**
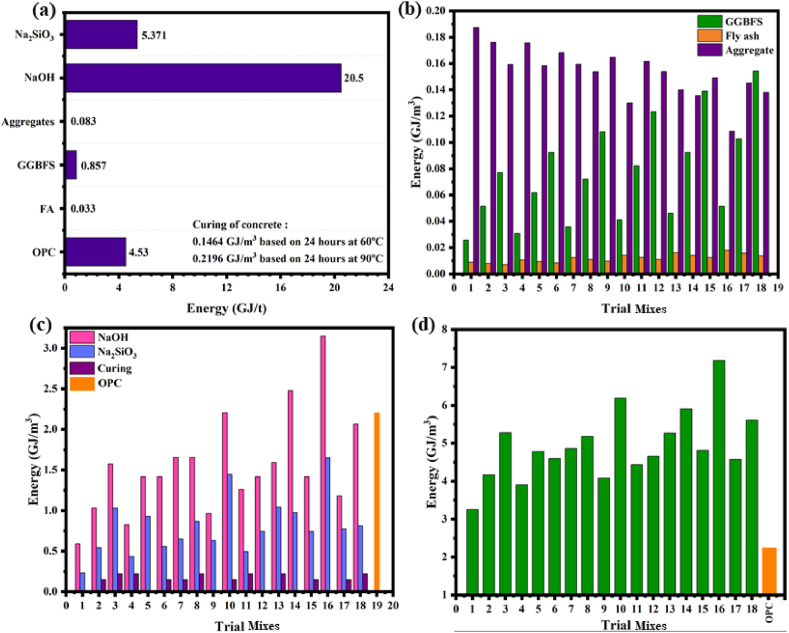
(a) the required energy of constituent materials; **(b & c)** the energy required by the constituent materials for 18 trails with reference to the energy required by OPC; **(d)** the overall amount of energy consumed for 18 trails with reference to the energy required by OPC.

The energy needs of several constituents, including industrial byproducts like FA, GGBFS, and RHA, are the basis for estimating the energy content of concrete. Depending on variables like FA, GGBFS, coarse gravel, fine gravel, and AA solution, different amounts of energy are required to produce 1 m^3^ of concrete [85]. A tonne of energy requires 0.033 GJ for FA and 0.857 GJ for GGBFS. For OPC, one tonne of cement requires 4.53 GJ of energy to produce. Fillers take up a substantial volume depending on their specific gravity and surface space of the material; they give stability and rigidity in both OPC and SC scenarios. One tonne of coarse aggregate and one tonne of fine aggregate require 0.081 and 0.083 GJ of energy, respectively. Moreover, AA has a major effect on the energy used to make concrete [86]. One metric tonne of NaOH and Na_2_SiO_3_ require 20.5 GJ and 5.371 GJ of energy, respectively [87]. The energy requirements of FA, GGBFS, and aggregates are 1 %, 7 %, and 6 %, respectively. The energy required by the constituent materials is depicted in Fig. 33(a).

Specifically, 56 % and 31 % of the energy are needed for AA solution. 1 m^3^ of OPC concrete requires 2.831 GJ of energy to make, and Fig. 33(b and c) displays the energy needed for eighteen trails. As can be shown in Fig. 33(d), the energy required to manufacture 1 m^3^ of FA GGBFS based SC increases with the quantity of GGBFS. The amount of energy consumed rises with the number of GGBFS. T_3_-5.279, T_6_-4.599, T_9_-4.088, T_12_-4.658, T_15_-4.816, and T_18_-5.614 GJ/m^3^ were the computed energy requirements for SC samples with varying GGBFS percentages. As the concentration of NaOH rises, the analysis finds that the system's energy needs increase proportionally. However, because the raw materials are close together, the energy needed for material transportation is not taken into account.

### 5.3 Eco-efficiency

The production of SC uses energy from various sources, including electricity, coal, and petroleum, for manufacturing and transporting components, resulting in CO_2_ emissions, which is compared to the energy consumption index [88]. According to estimates, the manufacture of OPC releases 0.73 to 0.85 t-CO_2_/t of CO_2_ per tonne; however, the CO_2_ emission is significantly lower for aggregate production and requires significantly less energy than the production of all other ingredients. Eco-efficiency can be used to describe the sustainability of FA-GGBFS blended SC with respect to CO_2_ emissions. The relationship between concrete's strength and CO_2_ emissions is used to assess the material's eco-efficiency [10,12,58]. Using the CO_2_ emission efficiency proposed by Alsalman et al. [85], a CO_2_ emission study for 1 m^3^ of concrete was carried out, and the results showed that each component of the concrete contributes to CO_2_ emissions. Nevertheless, Flower and Sanjayan [89] assessed CO_2_ emission estimates for FA, GGBFS, and aggregates but did not estimate NaOH and Na_2_SiO_3_, so the proposal does not provide the complete CO_2_ emission of SC.

**Fig. 34:**
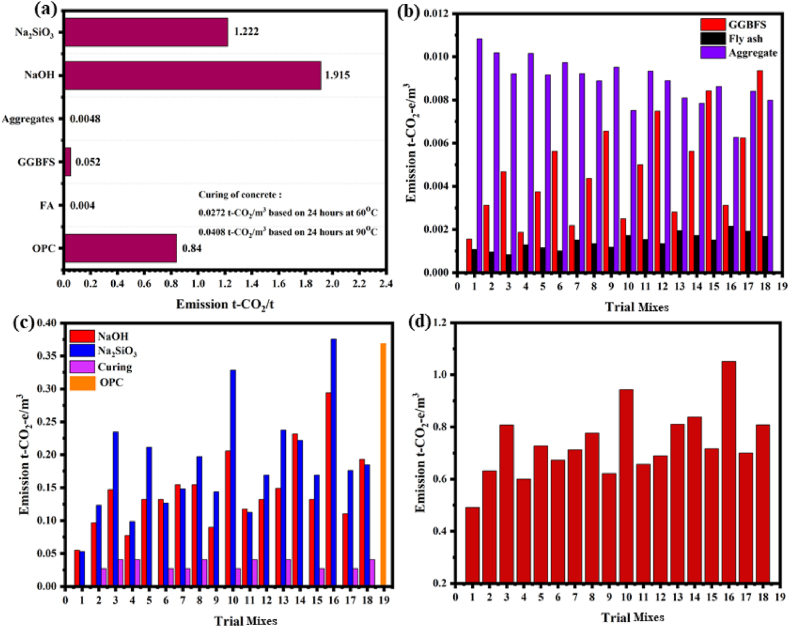
(a) the eco-efficiency of constituent materials; **(b & c)** the eco-efficiency by the constituent materials for 18 trails with reference to eco-efficiency by OPC; **(d)** the overall amount of eco-efficiency for 18 trails.

Heath et al. [90] and Hammond et al. [91] reported that the CO_2_ emission values for cement, GGBFS, and FA were 0.84, 0.052, and 0.004 t-CO_2_/t, respectively. For both fine and coarse aggregates, the CO_2_ release value is 0.0048 t-CO_2_/t. Additionally, Turner and Collins calculated the CO_2_ release value of binders, fillers, and AA solution. According to Turner and Collins [92], 100 % solid NaOH releases 1.915 t-CO_2_/t of CO_2_. As opposed to this, Na_2_SiO_3_ releases 1.222 t-CO_2_/t. As depicted in Fig. 34(a) NaOH concentration is directly proportional to carbon emission as well as energy consumption. Mix having lower NaOH concentration has less carbon emission than conventional cement-based concrete. As previously stated, this analysis did not take into account the CO_2_ emissions associated with shipping materials. The study analyzed the eco efficiency of FA GGBFS incorporated SC and OPC composites, finding a significant difference between the SC and OPC (Fig. 34(c–d)). The cost, energy, and eco efficiency of SC were also evaluated. The study concluded that replacing FA with GGBFS by 30 % leads to higher standards for hardness properties and workability in SC manufacturing.

## 6 Conclusions

The Taguchi-GRA approach can help determine the best control parameters for SC composition. This study assessed various parameters, including binder content, GGBFS content, NaOH molarity, AA ratio, and CT, on the slump, FD, DD, CS, FS, TS, MOE, IE, WA, and sorptivity of SC. The comprehensive evaluation provides insights into the multifaceted effects of different parameters on concrete performance. Statistical methods like ANOVA and SNR analysis are used for the optimization and prediction of concrete performance. The use of high-confidence regression equations indicates a rigorous approach to model development and prediction accuracy. The following conclusions are drawn from the results of this study.

i. GGBFS accelerates geopolymerization of FA-based SC, reducing setting time and early age CS. The presence of FA is also significant for proper setting time, workability, and CS augmentation at later ages. GGBFS additions slightly increased both fresh and hardened concrete densities, with a high correlation coefficient of 0.9057 enabling the prediction of hardened density.
ii. The CS of SC cubes exceeded 40 MPa, regardless of factors like AA ratio, CT, and NaOH molarity. The highest strength was observed in a trial mix with specific parameters: a binder content of 600 kg/m³, 30 % GGBFS content, 12 M NaOH molarity, 1.5 AA ratio, 0.35 AA to binder ratio, and a CT of 90 °C. Mixes with 10 % GGBFS content achieved a 28-day CS of over 30 MPa suitable for structural applications.
iii. Analysis reveals a direct relationship between the AA ratio and energy absorption, with WA and sorptivity most influenced by GGBFS content and binder content.
iv. The SNR graph was used to optimize parameter levels, with results tabulated. Design and optimization were conducted using contour and 3D surface plots. Regression equations were developed with over 95 % confidence, showing close agreement between experimental and predicted results.
v. SEM analysis showed that the optimal SC mix (T_18_) resulted in a dense C-A-S-H and N-A-S-H gel structure, while another mix (T_3_) had a more heterogeneous and porous structure. The sustainability index analysis indicated that the SC mix had lower cost and carbon emissions compared to OPC mixes, but slightly higher energy efficiency compared to OPC mixes.
vi. The study utilized GRA, ANOVA, and SNR methods to analyze properties varying by six variables. Results indicated that GGBFS content was the most influencing parameter, while the least influencing parameters varied with each method.

## 7 Implications of the research

⁃ The study demonstrates the optimization of various parameters on the characteristics of SC by advanced techniques such as Taguchi GRA, signal–noise ratio, and ANOVA. By assessing various parameters such as binder content, GGBFS content, NaOH molarity, AA ratio, and CT, the study provides insights into how these factors can be optimized to enhance the properties of SC.
⁃ The findings suggest that GGBFS accelerates the geopolymerization process of FA-based SC, resulting in reduced setting time and early age CS. This implies that incorporating GGBFS into SC mixes can lead to improved early-age performance and faster construction processes.
⁃ The study demonstrates that the cube CS of SC can exceed 40 MPa, meeting the requirements for structural applications. This suggests that the optimized SC mix developed in the study has the potential to be used in load-bearing structures, contributing to the advancement of sustainable construction practices.
⁃ The analysis reveals direct relationships between certain parameters (e.g., AA ratio, GGBFS content, binder content) and specific properties of SC (e.g., energy absorption, WA, sorptivity). Understanding these relationships can guide future research and practical applications in tailoring SC mixes to meet specific performance requirements.
⁃ The study utilizes advanced optimization and prediction methods such as SNR analysis, contour plots, and regression equations to optimize parameter levels and predict the total performance of SC mixes. These methods can be valuable tools for engineers and researchers in designing and evaluating SC formulations for different applications.
⁃ SEM analysis provides insights into the microstructure of SC mixes, indicating the formation of dense gel structures in the optimal mix. Additionally, the sustainability index analysis highlights the economic and environmental advantages of using SC over OPC mixes, reinforcing the importance of sustainable construction materials

## 8 Research limitations

⁃ While the study assessed various parameters such as binder content, GGBFS content, NaOH molarity, AA ratio, and CT, there may be other factors influencing the properties of SC that were not considered. For instance, factors like aggregate type and size, mixing method, and environmental conditions could also play a role in determining SC properties.
⁃ The study conducted experiments under controlled laboratory conditions, which may not fully represent real-world scenarios. Factors such as variability in materials, construction practices, and environmental conditions could affect the performance of SC in practical applications.

## 9 Recommendations for future research

⁃ Conduct long-term durability testing on the optimized SC mix to assess its performance under various environmental conditions and exposure scenarios. This could include testing for resistance to freeze-thaw cycles, chloride penetration, alkali-silica reaction, and other durability-related properties.
⁃ Investigate the effects of incorporating additional additives or supplementary cementitious materials on the properties of SC. For example, exploring the influence of silica fume, metakaolin, or slag on the performance and sustainability of SC mixes could provide valuable insights for further optimization.
⁃ Conduct a more detailed microstructural analysis, possibly using advanced imaging techniques such as transmission electron microscopy or X-ray diffraction, to further understand the relationship between microstructure and properties of SC. This could provide deeper insights into the mechanisms governing the performance of optimized mixes.
⁃ Further, optimize the sustainability aspects of SC mixes by considering additional factors such as resource depletion, water usage, and social impacts. This could involve exploring alternative materials, production methods, and transportation strategies to minimize environmental footprint and enhance social sustainability.
⁃ Compare the performance, sustainability, and cost-effectiveness of the optimized SC mix with other sustainable construction materials, such as high-performance concrete, recycled aggregates, or bio-based composites. This comparative analysis could help identify the most suitable materials for specific applications and environmental contexts.

## Data availability statement

Data will be made available on request.

## CRediT authorship contribution statement

**Samuvel Raj R:** Writing – review & editing, Writing – original draft, Investigation. **G. Prince Arulraj:** Writing – review & editing, Validation, Supervision, Methodology, Conceptualization. **N. Anand:** Writing – review & editing, Validation, Supervision, Methodology, Conceptualization. **Balamurali Kanagaraj:** Investigation. **M.Z. Naser:** Writing – review & editing. **Eva Lubloy:** Writing – review & editing, Funding acquisition.

## Declaration of competing interest

The authors declare that they have no known competing financial interests or personal relationships that could have appeared to influence the work reported in this paper.
